# Molecular Sexing and Species Detection of Antlered European Hunting Game for Forensic Purposes

**DOI:** 10.3390/ani12030246

**Published:** 2022-01-20

**Authors:** Petra Zenke, Orsolya Krisztina Zorkóczy, Pál Lehotzky, László Ózsvári, Zsolt Pádár

**Affiliations:** 1 Department of Animal Breeding and Genetics, University of Veterinary Medicine Budapest, István u. 2., H-1078 Budapest, Hungary; zorkoczy.orsolya@gmail.com; 2 Hungarian Hunters’ National Chamber, Medve u. 34-40., H-1027 Budapest, Hungary; lehotzkyfamily@gmail.com; 3 Department of Veterinary Forensics and Economics, University of Veterinary Medicine Budapest, István u. 2., H-1078 Budapest, Hungary; ozsvari.laszlo@univet.hu; 4 Department of Forensic Medicine, Medical School, University of Pécs, Szigeti út 12., H-7624 Pecs, Hungary; zsolt.padar@aok.pte.hu

**Keywords:** wildlife forensic genetics, molecular sex determination, species detection, red deer, roe deer, fallow deer, Amelogenin X/Y, SRY, cytochrome b

## Abstract

**Simple Summary:**

The reasons behind illegal hunting can be widely different. There are also tricky methods that hunters use in the attempt to legalize their illegally-acquired trophies, specifically, introducing them in later seasons, and registering the eviscerated corpses as hinds. During certain periods of the year, hunters are only able to acquire a license for the shooting of female deer exclusively, with the male-hunting season beginning later. The eviscerated and decapitated carcass of the animal delivered to the wild game processing house cold store can be falsely registered as a female based on phenotype. If the hunting association suspects that the animal’s sex has been falsely reported, a forensic genetic investigation can be proposed. In other criminal events, there are no carcasses at all. When several biological remains left on the crime scene need to be tested, a fast and cost-effective detection of a given species or species-group might be substantial before subsequent analysis. Therefore, sex and species detection can provide an early-stage credibility to the resolution of illegal activities related to trophy animals, and additionally may disclose potential poaching disputes.

**Abstract:**

Molecular sexing techniques are widely applied in conservation biology, although the range of forensically validated methods is fairly limited. The primary aim of this work was to develop forensically validated assays, using two PCR panels for sex and species assignment for the abundant antlered European game species: red deer (*Cervus elaphus*), roe deer (*Capreolus capreolus*) and fallow deer (*Dama dama*). Segments of the SRY and Amelogenin X/Y genes for sex determination, additionally species-specific cytochrome b regions for species detection were targeted and separately amplified in two multiplex reactions. These assays can reliably analyze trace amounts of DNA. The results of both can easily be visualized and interpreted practically, either on agarose gel or by capillary electrophoresis. These simple, fast molecular assays are able to affect the early-stage resolution of disputed or unsolved poaching cases, without the need of individualization or sequencing of forensic samples.

## 1. Introduction

Species identification and sexing methods have significant relevance in conservation biology, population ecology and also in wildlife forensics. It is reasonable that the genetic methods represent a major part of the current analytical toolbox for these tasks, particularly when partial, less recognizable, eviscerated remains of the animal are the single evidence for a given offense. This is not a rare scenario at wildlife crime scenes, especially in cases of traded illicit goods [1]. However, other molecular analysis, such as quantification of hormone ratios may also serve as an alternative approach [2]. In addition, several genetic markers for species determination or detection and sexing in different species have already been applied in a wide range of research fields [3,4,5,6,7,8].

The antlered game species usually play an important role in the ecosystem. Nevertheless, a considerable amount of abuse is connected worldwide to these animals in the past and present, and is expected to continue even in future [9,10,11,12,13]. The various deer species are also usually much-prized in Europe, although their economic importance can vary from country to country. Red deer (*Cervus elaphus*), roe deer (*Capreolus capreolus*) and fallow deer (*Dama dama*) are not only the most common game species in Europe, hunted for meat and trophies [14], but can also provide social values, even as mythological (“Wonder Deer”) or emblematic members (“Royal Game”) of the natural megafauna [15,16]. In several countries, such as Hungary, there are limits on their harvest as game animals. There is a limited time where they are available for harvest and additionally related hunting laws are usually specific as to the sex of the animal being culled [15,17,18]. In addition, according to the hunting area or hunting companies, shooting prices can be different, depending on whether they are labeled as male or female [19,20].

Unlike human forensics, the genetic evidence is differently focused on source and sub-source level in the field of wildlife forensics. Species detection and sex determination can be crucial to determine whether a crime has occurred, or not [1]. In cases in which the hunting law, hunting areas or timeframe are specific to the species and/or sex of the harvested animal, the process of individualization is not always required. When a suspicion arises that a trophy or a particular carcass does not match with the given eviscerated body or the data on the documentation, forensics frequently have to provide only single sex determination without genetic profile.

Selective hunting for bigger trophies has been accompanied by the importance of a trophy hunting industry [21]. For example, in Hungary the official tabs fixed on the animal corpses can identify legally hunted and documented animals in a processing house but cannot prevent the cheating cases with trophies. The eviscerated body of illegally harvested (taken out of legal timeframe) antlered gender could be introduced as the official tab as a female form in a given time (Figure S1). The promising trophy would be registered months and even years later than when the shooting period of the male gender has officially been made legal. The latter forensic investigation and prosecution of these types of cases usually require more complex examination, though the fast and cost-efficient gender-screening of eviscerated carcasses not only may solve alleged offences, but also can increase the prevention. Wildlife forensic investigations often are undertaken by academic institutions without forensic quality assurance standards [22,23,24]; therefore, there is growing demand for forensically validated assays.

Over the years, a large number of sequences on sex-specific sequence variants have been used for various deer species in conservation biology [6,25,26,27,28,29,30,31]. These studies. while providing a good foundation, do not meet the criterion set by forensic science [23,32,33]. In spite of the efforts in related research, this field is quite heterogeneous, and the range of forensically validated assays are relatively limited [6,34,35,36]. Although, recently developed marker sets for forensic applications usually incorporate genetic markers for sex determination [14,15,17], there are only few published validated assays simultaneously detecting sex and species on cervids [31].

On many occasions, there are no carcasses at all, only biological remains left on the crime scene (e.g., snare looping, poaching cases, transfer or trade, traffic accidents caused by game). In these cases, the identification or detection of a given species might be substantial to make a decision in a legal dispute [1,24]. In contrast to human forensics, the species identification in wildlife cases can be subject to unique requirements and considerations [8,37,38]. Determining the origins of non-human biological material as ‘silent witness’ found on the belongings of the suspects and/or at crime scenes can increase the possibility of identifying the culprit [12,13,39,40,41].

Several recent methods of sex determination have been developed for the detection of single Y-chromosome specific sequences, completed with nuclear or mitochondrial internal controls to confirm the adequacy of reaction [12,25,26,27,28,29,30]. However, the absence of a signal male specific marker does not necessarily mean that the sample is of female origin. The allelic drop-out caused by sample degradation or technical imbalances can also lead to false negative results [35,42]. To avoid this type of misinterpretation, the simultaneous detection of at least two different Y-chromosome specific sequences is the most reliable method. This is the case not only for humans or primates, but for other species as well. A complementary sexing protocol developed for detection of the X and Y chromosome linked Amelogenin gene or STR markers can disambiguate the confirmation of analytical results on the SRY gene [43,44].

For species identification, two genetic markers on the mitochondrial genome are mostly analyzed by sequencing: the cytochrome oxidase I (COI) gene, which is the basis of DNA barcoding initiative and BOLD database (Bold Systems v4; http://boldsystems.org/, accessed on 22 November 2021.) and the cytochrome b (Cytb) gene. Both meet the criteria for species assignment, such as high inter-species variability and low intra-species variation [45], and therefore commonly applied in forensic cases [12]. Multiplexing several primers together in one reaction mixture enables simultaneous identification of target species and improves the success rate of species detection when using only a minute amount of DNA. In contrast to the sequencing-based methods, combining species-specific primers with fragment length analysis, real-time PCR detection or multiplex hybridization assay allow the parallel identification of more species and can be applied to a DNA mixture [3,46,47,48,49]. As many crime scenes are outdoor “living crime scenes”, evidential samples are often exposed to environmental breakdown, and DNA shows considerable degradation over time, which can inhibit the subsequent DNA analysis. Success of evaluating trace amounts of DNA depends on the length of the amplified fragment. Methods, using shortened amplicons are able to significantly increase the success rate of analysis from degraded remnants [50].

Although a combination of rapid molecular assays for species detection and sex determination in the case of swamp deer (*Rucervus duvaucelii duvaucelii*) has previously been described for conservation purpose [31], the criteria of forensic validation as well as extending of testing to European deer species are not mentioned or referred to. Red deer (*Cervus elaphus*), fallow deer (*Dama dama*) and roe deer (*Capreolus capreolus*) are not only among the most important game species in European and Near Eastern countries, having a wide distribution in many regions, but they can also be found on other continents. The objective of this work was to create a relatively fast and validated assays for molecular sexing and species detection for these three above mentioned species, which is suited for simultaneous visualization by capillary electrophoresis (CE) and is easy to use in poaching or illegal trading cases.

## 2. Materials and Methods

### 2.1. Sample Collection and DNA Extraction

All samples, such as muscle, hide, hair and feces were collected from registered shootings of the given species. Red deer (*n* = 30), fallow deer (*n* = 47) and roe deer (*n* = 38) with known sex were provided by the Pilis Park Forest Hunting Company and by hunters with a license (Table S1, Figure S2).

Genomic DNA was isolated from the muscle, hide and hair samples using a DNeasy^®^ Blood and Tissue Kit (Qiagen, Hilden, Germany), and a QIAamp^®^ Fast DNA Stool Mini Kit (Qiagen, Hilden, Germany) was used for the feces samples, following the procedural guidelines as instructed. Extracted DNA quality was measured on 1% agarose gel using a GelRed^TM^ Nucleic Acid Gel Stain (Biotium, Fremont, CA, USA), and concentration was measured by a Qubit 2.0 Fluorometer (Life Technologies Corporation, Carlsbad, CA, USA). Extracted DNA from the tissue samples were stored at 4 °C until subsequent analysis.

### 2.2. Marker Selection and Primer Design

DNA sequences of Sex-determining Region Y (SRY), Amelogenin X and Y (AmelX/Y) and cytochrome b (Cytb) genes of the three species were obtained from the NCBI GenBank database (https://www.ncbi.nlm.nih.gov/genbank/, accessed on 28 November 2021).

The selected homologue sequences of SRY, Amel X, Amel Y and Cytb genes were aligned in MEGA (Molecular Evolutionary Genetics Analysis across computing platforms) [35] to identify conservative regions of similarity in the case of AmelX, AmelY and SRY genes. In case of the Cytb gene, the species-specific SNP (single nucleotide polymorphism) variations were identified and compared for developing species-specific forward primers, the reverse primer was designed universally for all the three species. For gender analysis, the universal primers were created for the three given species using the conserved parts of the AmelX, AmelY and SRY markers. All primers were designed by Primer Designer 4 software (http://www.scied.com, accessed on 28 November 2021). Selected primers have the length of 18–36 bases and no self-complementarity (hairpins) or complementarity to other primers (more than four base pairs at the 3′ end) were observed. Moreover, the primers were designed considering the reduced amplicon size of <220 bp to assure higher amplification success and the varying amplicon sizes among markers enable the parallel detection with capillary electrophoresis (Table 1).

In order to examine possible cross-amplification with the goal of discovering limitations or widened utilization fields, DNA sequences of each marker from a great variety of related cervid and other species were obtained from the NCBI GenBank database (https://www.ncbi.nlm.nih.gov/genbank/, accessed on 28 November 2021) for further in silico analysis. Intra-population diversity at the primer binding sites for each marker and investigated species were also tested by MEGA platform.

### 2.3. Singleplex Reactions and Sequencing Analysis

Amelogenin, SRY and cytochrome b gene fragments were amplified with male and female red deer, roe deer and fallow deer samples in singleplex reactions to determine functionality. PCR (25 μL in volume) containing 12,5 μL DreamTaq™ Green PCR Master Mix (ThermoFisher Scientific, Waltham, MA, USA), 1 μL of BSA (10 mg/mL, Sigma–Aldrich, St. Louis, MO, USA), 0.5 μM unlabeled forward and 0.5 μM unlabeled reverse primers, 5 ng DNA template and PCR grade H_2_O to volume. PCR was carried out in an AppliedBiosystems 2720 Thermal Cycler with the following conditions: an initial 95 °C for 30 s followed by 36 cycles of 25 s at 94 °C, annealing of 25 s at the given marker specific temperature (as described in Section 2.4) and 25 s at 72 °C, and final extension for 20 min at 72 °C, for subsequent sequencing reactions. Amelogenin X and Y alleles were separated on 2.5% agarose gel, the selected allele bands were cut from the gel and purified with Gel Advanced^TM^ Gel Extraction System (Viogene, Sunnyvale, CA, USA). The reamplification parameters were the same as singleplex PCR (see above) using 26 cycles. All PCR amplicons were purified using GenElute^TM^ PCR Clean-Up Kit (Sigma–Aldrich, St. Louis, MO, USA). Both DNA strands were sequenced using the BigDye^®^ Terminator v.1.1 Cycle Sequencing Kit (Thermo Fisher Scientific, Waltham, MA, USA) in the manner recommended by the manufacturer. For sequence detection, an ABI Prism 3130XL Genetic Analyzer (Applied Biosystems, Waltham, MA, USA) was applied, according to manufacturer’s guidelines. Sequence analyses were performed using Sequencing Analysis Software 5.1 (Applied Biosystems, Waltham, MA, USA) and aligned by Sequencher^TM^ 4.1.2 software (Gene Codes Corp, Ann Arbor, MI USA). A homology search of resulted sequences was accomplished using the GenBank (BLAST^®^, Basic Local Alignment Search Tool).

### 2.4. Multiplex Amplification and Detection

Annealing temperatures were optimized for a diplex use of the sex determining loci, hereafter referred to as “*DeerSex-plex*”, and in a separate triplex reaction for the species detection markers, hereafter referred to as “*DeerSpec-plex*”. PCR conditions were optimized as follows, using female and male genomic DNA obtained from each of the three above mentioned species. The primer concentrations were combined equimolar first, then based on the peak heights of the capillary electropherograms, variable concentrations were set to obtain inter-locus balance (Table 1). Negative (no-template) reaction controls were applied in each step of analysis to avoid/detect the risk of contamination.

Amplification of AmelX/Y and SRY were performed in *DeerSex-plex* in a 10 μL reaction volume, containing 5 μL DreamTaq™ Green PCR Master Mix (Thermo Fisher Scientific, Waltham, MA, USA), 0.5 μL of BSA (10 mg/mL), 1.5 μL of primer-mix, 2 ng of DNA template and PCR grade H_2_O to volume. Touch-down PCR was carried out on Applied Biosystems 2720 Thermal Cycler using the following conditions: an initial step at 95 °C for 30 s followed by 20 cycles of 20 s at 94 °C, annealing of 20 s starting at 54 °C and decreasing by 0.2 °C per cycle and 20 s at 72 °C, followed by 14 cycles of 20 s each at 95 °C, at 50 °C, at 72 °C and final extension for 20 min at 72 °C.

For species detection, three specific forward primers and one universal reverse primer were designed to amplify the Cytb gene segment in a 10 μL reaction volume in *DeerSpec-plex*, containing 5 μL DreamTaq™ Green PCR Master Mix (ThermoFisher Scientific, Waltham, MA, USA), 0.5 μL of BSA (10 mg/mL), 1.5 μL of primer-mix (three species-specific forward and the universal reverse primer), 2 ng of DNA template and PCR grade H_2_O to volume. PCR was carried out on Applied Biosystems 2720 Thermal Cycler using the following conditions: an initial step at 95 °C for 30 s followed by 34 cycles of 25 s at 94 °C, annealing of 25 s at 64 °C and 25 s at 72 °C, and final extension for 20 min at 72 °C.

From both (sex- and species detection) assays, the amplified products were separated and visualized on a 2% agarose gel using GelRed^®^ nucleic acid stain (Biotium, Fremont, CA, USA). The two reactions were then analyzed by capillary electrophoresis on an ABI Prism 3130XL Genetic Analyzer using GeneScanTM-500 LIZ^TM^ Size Standard (Thermo Fisher Scientific, Waltham, MA, USA). The minimum detection threshold was set at 150 relative fluorescence unit (RFU) during fragment analysis using GeneMapper^®^ ID-X software version 1.4. Bin-width used was 1 bp, which was set for allele designation for each investigated marker based on allele sizing of sequencing verified alleles.

### 2.5. Basic Validation

To estimate the reliability and limitations of the two assays, the steps of basic developmental validation were implemented according to recommendations [32,51].

Cross-species amplification of both plexes (*DeerSex-plex* and *DeerSpec-plex*) were tested on male and female samples (input amount of 2 ng) from related species such as cattle (*Bos taurus*), takin (*Budorcas taxicolor*), bison (*Bison bison*), mouflon (*Ovis musimon*) and sheep (*Ovis aries*); and on samples from forensically relevant species such as human, swine (*Sus scrofa*), dog (*Canis lupus familiaris*) and cat (*Felis catus*). The success rate of intra-species amplification was tested by both plexes on 115 samples—red deer (*n* = 30), fallow deer (*n* = 47) and roe deer (*n* = 38)—collected from different regions of Hungary.

Sensitivity was evaluated using 1.0, 0.5, 0.25, 0.125 and 0.062 ng DNA templates in each reaction. Each DNA dilution was amplified with both assays two times on different days using the same dilution series.

Specific alleles of the sensitivity and reproducibility studies detected by CE were used for calculating peak height ratios. Heterozygote balance in the case of AmelX and AmelY marker in male animals was calculated by dividing the peak height of the higher peak by the peak height of the lower peak.

Both plexes were tested on forensically relevant sample types, such as muscle, hide, hair and feces. Five previous casework DNA samples, including blood stain, blood swab and hairs from different poaching cases involving red deer and fallow deer collected in 2019 and 2020, were reanalyzed. Conditions of DNA preparation and amplification are described in Section 2.4.

For the study mixture, equal DNA amounts (1-1 ng) were used to prepare the red/roe/fallow deer DNA mixture. Additionally, 5 ng human DNA was also mixed with 0.5, 0.25, and 0.125 ng red-, roe-, and fallow deer DNA in separate tubes. These mixtures were examined with the DeerSpec-plex assay described in Section 2.4. To estimate the sensitivity of male specific markers’ detection 5 ng female red-, roe-, and fallow deer DNA were mixed with 1, 0.5, and 0.33 ng male red-, roe-, and fallow deer DNA in separate tubes and examined with the *DeerSex-plex* assay described in Section 2.4.

The accuracy was established through repeatability and reproducibility studies. The multiplexes were tested by one operator on two runs, and by different operators on separate days on two runs.

### 2.6. Field Samples—Mock Cases

In two cases, five field samples—blood, vessel, muscle, bone and hair samples—from two red deer corpses were provided by two official veterinarian examiners of Hungarian hunting companies (Figure S3). The preliminary hypothesis in both cases was that the samples had to come from red deer hinds, which were delivered into processing houses.

## 3. Results

DNA was successfully extracted, and measurable amounts (0.8–96.4 ng/μL) of DNA were obtained from blood stain, muscle, hide, hair and feces.

### 3.1. Marker Selection and Primer Design

The SRY and AmelX, AmelY loci were selected for sex determination and four primers were designed to amplify both gene regions for the three species of interest. One reverse primer and three species-specific forward primers based on the SNP pattern of the Cytb gene of red deer, roe deer, and fallow deer has been created for species detection (Table 1). Intra- and inter-species specificity were estimated using as many sequences as available from GenBank for in silico analysis (Table S2, Figures S4–S6). Regarding the four sexing primers (SRY forward and reverse, AmelX/Y forward and reverse), only one SNP position within the AmelX/Y reverse primer was observed in the downloaded sequences (in roe deer species). Within the Cytb reverse primer, only one SNP affected the primer binding site in fallow deer and two SNP in red and roe deer samples. The forward primer sequence of *Capreolus capreolus* Cytb contains three possible SNPs from 135 downloaded roe deer sequences. However, based on 435 downloaded red deer sequences, a relatively high number of polymorphic base positions (nine SNPs) can be existing within the Cytb *Cervus elaphus* forward primer binding site, five of them have only <1% frequency. Within a single sequence, the most common SNP numbers were two in primer binding sites of the Cytb marker in red- and roe deer samples. No SNP positions were observed at the 3′ end position within the six developed primer sequences from neither species.

Having sequences available from a great variety of species found in GenBank, similarities among species were found based on the in silico analysis using MEGA software (Figures S4–S6). The results show that the designed primers for sexing could be used in several other cervid species in the case of SRY and Amelogenin X/Y genes (Figures S4 and S5).

### 3.2. Singleplex Reactions, Sequencing and GenBank Submission

The PCR products from each marker were confirmed by sequencing. The sequences were submitted into GenBank (accession no. MW876187-MW876198), which indicated homology with the Amelogenin X and Y, SRY and Cytb genes of red deer, roe deer and fallow deer. Possible SNP positions within the primer sequences can cause a partial homology in these species, but the specificity of the result was not influenced. Primers do not require full matching in the middle section (Table 1).

### 3.3. PCR Based Multiplex Design and Detection

**Figure 1:**
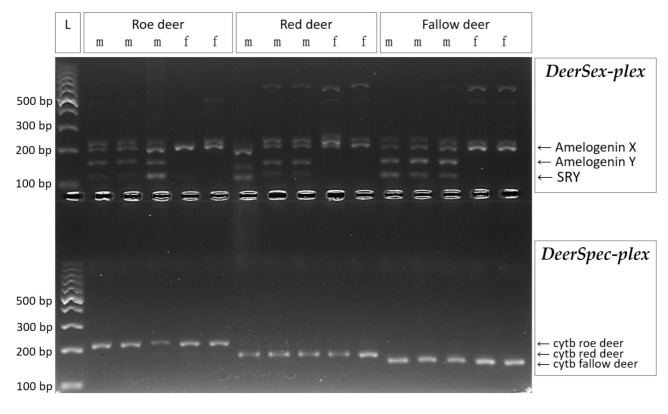
Multiplex PCR amplification for three cervid species. *DeerSex-plex* panel contains Amelogenin markers (194 bp for X and 140/149 bp for Y allele) and SRY (113 bp), the males show three specific bands and females show a single specific band in the agarose gel. *DeerSpec-plex* panel contains species-specific cytochrome b markers for roe deer (218 bp), red deer (176 bp) and fallow deer (162 bp) and show a single species specific band on agarose gel. L: ladder, m: male sample, f: female sample.

Our original goal was to amplify all selected markers for sex- and species detection in one multiplex reaction, but this attempt failed due to the significant differences among the optimal annealing temperatures, which induced allele/locus drop-out and artefact peaks. Accordingly, primers of sex- and species detection markers were developed and optimized successfully in two separate multiplexes. The specific fragments in question were robustly amplified and detected on agarose gel from control samples with known sex- and species controls (Figure 1). As expected, both Y-chromosomal (SRY and AmelY) and X-chromosomal (AmelX, as a reaction control) segments were detected in male samples, while only the AmelX was detected in female samples using the *DeerSex-plex* assay. Although some aspecific fragments were also produced, the artefacts did not influence the judgment of the sex, as their sizes are longer than the specific Amelogenin X allele.

All of the three species-specific primers in the *DeerSpec-plex* designed for the cytochrome b gene amplified only the target cervid species using the optimized multiplex PCR profile (Figure 1). To avoid cross species or nonspecific amplifications during testing, annealing temperature should reach 64 °C.

**Figure 2:**
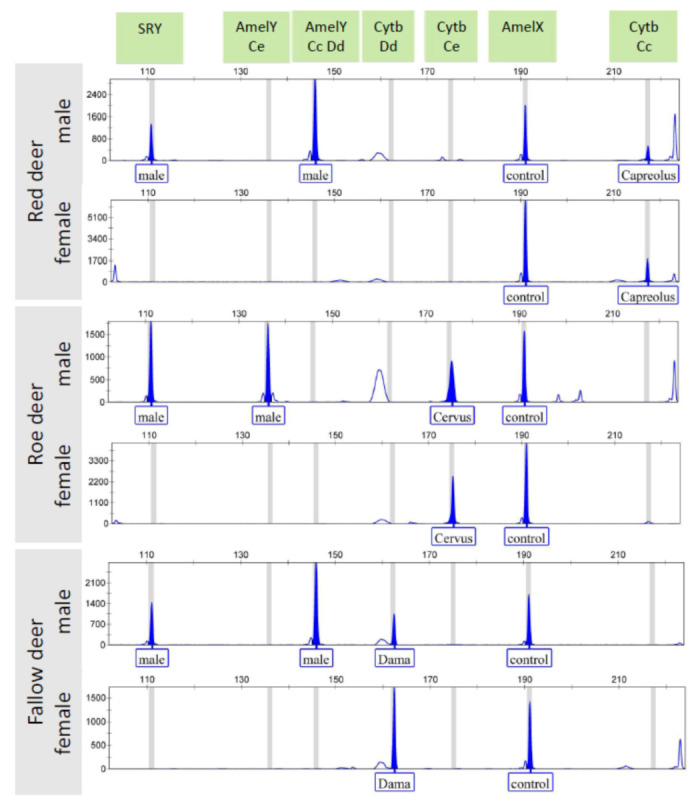
Parallel capillary electrophoresis detection of the PCR products amplified separately by *DeerSex-plex* and *DeerSpec-plex* assays for three cervid species. AmelY Ce: Amelogenin Y marker specific for red deer, AmelY Cc Dd: Amelogenin Y marker specific for roe deer and fallow deer, Cytb Dd: cytochrome b marker specific for fallow deer (*Dama dama*), Cytb Ce: cytochrome b marker specific for red deer (*Cervus elaphus*), Cytb Cc: cytochrome b marker specific for roe deer (*Capreolus capreolus*).

Due to the different amplicon lengths, followed by PCR amplification and agarose gel electrophoresis, a parallel detection of each PCR product from both assays was feasible by capillary electrophoresis using fluorescent labeled primers with 6-FAM dye (Table 1, Figure 2). The CE detection can be implemented in optionally mixed form, in which based on the strength of the amplicons’ bands on the semiquantitative agarose gel an approximate 1:1 mixing ratio of the two plexes can be appropriate. The males show two Y-chromosomal peaks (SRY, AmelY) and the AmelX as a reaction control, plus a species specific peak (Cytb); females show a single sex chromosomal peak (AmelX) as a reaction control, plus a species specific peak (Cytb). Based on the size of the amplified cytochrome b gene, the species origin can be clearly determined. Some artefact peaks were also detected out of the bin sets, these did not influence the reliability of the evaluation of results.

As expected, no-template reaction control samples were negative in each reaction by gel electrophoresis and by capillary electrophoresis.

### 3.4. Developmental Validation

#### 3.4.1. Species Specificity

Species specificity was separately tested on male and female samples in the case of the *DeerSex-plex* to know whether detected specific or aspecific PCR products have relatedness with the sex, and on female samples regarding the *DeerSpec-plex* assay (Figures S7 and S8).

*DeerSex-plex* assay: in the case of mouflon and sheep, three amplicons were detected at SRY, AmelY and AmelX bin positions on male samples, and one amplicon was detected at AmelX bin on female samples. No specific AmelX allele was observed using samples from other species, neither from males nor females. Using male cattle sample, PCR product at SRY and AmelY bin could be found specifically to the male sex. Using bison, dog and cat samples, PCR product at SRY bin could be found specifically to the male sex, as other amplicons at AmelY bin position or out of range could be observed on female samples as well. In cases of human, swine and horse only, one amplicon fell into (or very close to) bin position of AmelY marker, but from both sexes. No amplicons within the specified bin sets on the capillary electropherogram were observed using takin DNA, neither from the male nor female animal (Figure S7).

*DeerSpec-plex* assay: The designed primers for cytochrome b gene segments showed limited species specificity since two PCR products manifested very close to the marker specific bin settings in the case of the mouflon and sheep, and one amplicon using bison DNA (Figure S8). However, no PCR product has been detected using human, cat, horse, swine, takin, cattle or dog DNA.

Intra-species specificity study based on the analysis of collected population samples shows that all investigated samples amplified with *DeerSpec-plex* provide a clear PCR product on agarose gel (Figure S9). Using the *DeerSex-plex* assay, all but one sample (Dd_16) could be effectively determined for the sex by agarose gel detection (Figure S10).

#### 3.4.2. Sensitivity, Mixtures and Case-Type Samples

Sensitivity of multiplexes were evaluated using template concentration in dilution interval of 1 to 0.06 ng. Clearly visible specific bands were observed in each three species until 0.125 ng dilution using the *DeerSpec-plex* assay and until 0.5 ng dilution using the *DeerSex-plex* assay. Below this DNA amount, AmelX/Y or SRY locus drop-out could be observed (Figures S11 and S12).

Using DNA mixtures from red deer, roe deer and fallow deer, all three species could be detected until 0.06 ng dilution using the species identification assay. The detectability of male specific markers proved to be sensitive enough to see them on agarose gel using 5 ng female and 0.33 ng male red and fallow deer DNA. In the case of roe deer, 5 ng female and 0.5 ng male DNA gave a visible result of each allele involved in *DeerSex-plex* assay (Figure S13).

Extracted DNA from blood swab, blood stain, hairs, muscle and hide could be successfully amplified, resulting specific amplicons clearly visible on agarose gel and on capillary electropherogram as well (Figures S14–S16).

The electropherograms obtained in the course of the sensitivity study provided the basis for the calculation of intra-locus peak height ratios. The peak height ratios for AmelX/Y ranged from 0.66, 1.11 and 1.51 to 9.26, 2.29 and 11.26, with an average value of 2.35, 1.67 and 3.85 in roe deer, red deer and fallow deer (respectively).

#### 3.4.3. Field Samples—Mock Cases

The field sample (vessel) in case 1 resulted in red deer specific Cytb, and male-specific fragments, so the initial hypothesis, namely the sample originated from a female was excluded (Figure S15). The field sample (corium) in case 2 resulted in red deer specific Cytb, and female-specific fragments, so the initial hypothesis was supported by analysis (Figure S16).

## 4. Discussion

Poaching is a criminal activity, which is strictly defined by laws regulating the taking of wildlife. The literature is filled with studies of poaching cases worldwide, since the illegal killing of wildlife has been a social problem for centuries [10]. Parallel with the changes in law, new, highly reliable, molecular techniques and markers for sex- and species differentiation are also needed. In compliance with the SWGDAM [51], SWFS [52] and ISFG [32] recommendations, standards and guidelines for animal and wildlife forensic analysis, these methods can be incorporated into forensic practice only when systematic validation steps are performed.

For this purpose, we set up and validated two assays for sexing- and for species assignment, which generated reproducible and reliable data from a range of sample types. The validation process regarding the sensitivity, specificity, mixture study and repeatability was accomplished to the forensics standards; therefore the assays are applicable to the enforcement for the detection of both species and sex for red-, roe- and fallow deer species.

The sexing panel simultaneously amplifies two conserved regions of the Y-chromosome, the SRY and the Amelogenin Y gene, and one X-chromosomal locus (AmelX) providing an internal control. Double testing of Y-chromosome loci provides a result with higher accuracy, thus decreasing the chance of a false-positive or false-negative result. The assumption that the Amelogenin gene exists on both X and Y chromosomes with a deletion region on the Y chromosome in these species was supported by our results, as DNA fragments of different length between the male and female animals can be detected. As only red deer from the three tested species has a nine base pair deletion within the amplified AmelY region (Table 1, Figure 2), the sexing assay by itself can also indicate the species origin. However, based on GenBank sequences, this deletion exists also in Sika deer (*Cervus nippon*). In addition to the investigated cervid species, specific amplicons were detected from the nearest taxonomically related species (mouflon and sheep) for each marker, but with quite low signal intensity at the AmelY marker. However, since some other minor PCR products outside of the bin settings were also manifested based on sequences aligned in MEGA, the possible utilization may extend to these species and other cervid species as well (Figures S4–S6). Major artefacts affecting the interpretation of the sex were not observed.

Amplifying short mitochondrial DNA regions with species-specific primers, using different point mutations within the forward primer 3′ binding sites, is an effective method for simultaneous detection of mammalian species [3]. Species-specific primers can be designed based on inter species SNPs, provided those SNPs have little or no intraspecies variation [53]. In this study, all the four sexing primers (AmelX/Y forward and reverse, SRY forward and reverse) and the *Cytb Dama dama* forward primer proved to be designed to a highly conserved region within species, however limited number of GenBank sequences were accessed (Table 1 and Table S2). Although within the Cytb primers binding sites one to four SNPs can occur, drop out of specific fragment was not observed in our study (Figure S9). In cases where there is a well-defined investigational question i.e., a nominated set of well-characterized species, an assay using primers to target SNPs can be a cost-effective and rapid alternative to sequencing, followed by agarose or/and capillary electrophoresis [1].

Based on this assumption, there have been multiplex tests developed to detect a small number of deer species [54,55]. As it cannot be excluded that unforeseen species may produce a signal that is similar in length but not homologous with the target taxon, these tests are often considered presumptive methods of detection and not identification [56]. These markers, however, can be used as a screening tool to categorize samples for sequencing and are particularly suited for situations where sequencing commonly fails, such as when species mixtures are expected (e.g., common food meats or animal based traditional medicine) [50,57,58]. Such assays are best suited to situations where there is a limited number of expected species; they also require more extensive validation by sequencing to ensure the specificity of results [56].

Among ungulates, the evidence of hybridization varies in different countries [59,60,61,62,63]. The volume and effects of anthropogenic hybridization and wildlife translocations can differ region to region, as well as the efficiency of efforts of wildlife conservation or national prevention [21]. However, where there is a possibility of the presence of hybrids, the single utilization of maternal inherited mt DNA marker is not appropriate. The limitation using mitochondrial genes for species assignment can be solved with the expanded examination tool of paternal and/or biparental markers, for instance microsatellites or SNPs [63,64,65,66,67,68]. However, it is noted that the determination of species origin of hybrids requires more complex genetic methodology involving statistical analysis [69,70].

In our developed *DeerSex-plex* and *DeerSpec-plex* assays, the different fragment sizes were well separated in agarose gel electrophoresis; thus, different sex and species origin were easy to identify. However, fluorescent gel electrophoresis adaptation was implemented for better resolution by labeling the PCR products with fluorescent dyes. Combining the two panels in one capillary electrophoresis detection gives a rapid and prompt result (attention should be paid to use a proper mixing ratio of the two reactions) and makes sample identification more reliable and cost-effective. These approaches facilitate fast screening of evidential samples of various qualities collected from crime scenes, reduce time and cost in comparison with traditional sequencing or restriction fragment length polymorphism-based methods. Such molecular approaches are definitely advantageous when there is a need to test several samples for whether they come from the relevant species or sex in connection with the given case. In this study, we obtained no misidentification regarding the sex or species of the three tested cervid species; however, some imbalance among the specific peaks as well as some non-specific peaks could be observed.

## 5. Conclusions

Simple and precise methods for sex and species assignment in animals are a prerequisite for several applications in animal production, wildlife conservation and forensics. This study targeted species and sex detection for forensic purpose of three coexisting European cervid species as hunting games. Our methods, which match to forensic standards, could contribute credibility to the resolution of potential poaching disputes and confine the illegal activities related to red deer, roe deer and fallow deer.

## Figures and Tables

**Table 1:** Details of the sex chromosome and species-specific markers used.

Marker	Primer Sequences 5′-3′ and Fluorescent Dye	Primer μM	Size in Base Pair (by Species)	GenBank Accession Numbers
AmelX/Y	F: 6-FAM_CAACACCACCAGCCAAAC	1	X: 194 (Ce, Dd, Cc)	MW876193-MW876195
	R: AATATcGGAGGCAGAGGT		Y: 140 (Ce) 149 (Dd, Cc)	MW876196 MW876197-MW876198
SRY	F: 6-FAM_TCAGCAAGCAGCTGGGGTAT	0.4	113 (Ce, Dd, Cc)	MW876187- MW876189
	R: ATAGCCCGGGTATTTGTCTC			
Cytb	F: GGgGCATCAATatTTTTcATcTGtcTgTTtA	0.5	176 (Ce)	MW876190
	F: CTTTATCTGCCTATTCATCCATGTT	0.5	162 (Dd)	MW876191
	F: GACGTcAACTATGGtTGAATTATCCGATAtATACAT	0.5	218 (Cc)	MW876192
	R-univ: 6-FAM_GTTGCTCCTCAaAATGATATTTGTCC	1.5		

F: forward primer, R: reverse primer, R-univ: universal reverse primer for Cytb gene of the three species (lower case within the primer sequence indicates possible polymorphic nucleotide positions), bp: base pairs detected in females (X) and males (Y) in different species, Ce: *Cervus elaphus*, Dd: *Dama dama*, Cc: *Capreolus capreolus*.

## Data Availability

The data will be available with the corresponding author upon request.

## Supplementary Materials

The following are available online at https://www.mdpi.com/article/10.3390/ani12030246/s1, Figure S1: Eviscerated and decapitated carcasses in processing house cold store. Table S1: Sample database from three antlered species used for validation studies. Figure S2: Distribution of collected samples within Hungary from three antlered species. Figure S3: Field sample types. Table S2: In silico intra-species variation study of developed primers. Figure S4: Conservative regions of the SRY gene identified among species by MEGA. Figure S5: Primer binding regions marked out with yellow color within the highly conservative regions of the AmelogeninX/Y gene among species identified by MEGA. Figure S6: The species-specific forward and the universal reverse primer binding regions within the cytochrome b gene among species identified by MEGA. Figure S7: Species specificity tested on male and female samples using the DeerSex-plex assay, detected on agarose gel and by capillary electrophoresis. Figure S8: Species specificity tested on female samples using the DeerSpec-plex assay, detected by agarose gel and capillary electrophoresis. Figure S9: Intra-species specificity study, population samples from three species amplified with *DeerSex-plex* and detected on agarose gel. Figure S10: Intra-species specificity study, population samples from three species amplified with *DeerSex-plex* and detected on agarose gel. Figure S11: Sensitivity tested on male and female samples using the DeerSex-plex and DeerSpec-plex assays on agarose gel. Figure S12: The SRY and AMELX/Y loci in red (a), roe (b) and fallow (c) deer samples amplified with varying amounts of input DNA with the DeerSex-plex assay. Figure S13: Mixture study: red deer, roe deer and fallow deer samples amplified with varying amounts of input male DNA with the DeerSex-plex assay. Figure S14: Case type samples amplified with both assays. Figure S15: CE result of field sample (vessel) in mock case 1. Figure S16: CE result of field sample (corium) in mock case 2.

## References

[1] Moore M.K., Frazier K. (2019). Humans Are Animals, too. Critical Commonalities and Differences Between Human and Wildlife Forensic Genetics. J. Forensic Sci..

[2] Blecher A.S., Ganswindt A., Scheun J. (2021). Scales of our lives: Sex identification of Temminck’s pangolin (*Smutsia temminckii*) using scales retrieved out of the illegal wildlife trade. Gen. Comp. Endocrinol..

[3] Tobe S.S., Linacre A.M.T. (2008). A multiplex assay to identify 18 European mammal species from mixtures using the mitochondrial cytochrome b gene. Electrophoresis.

[4] Pereira F., Carneiro J., Matthiesen R., van Asch B., Pinto N., Gusmão L., Amorim A. (2010). Identification of species by multiplex analysis of variable-length sequences. Nucleic Acids. Res..

[5] Ramón-Laca A., Linacre A.M., Gleeson D.M., Tobe S.S. (2013). Identification multiplex assay of 19 terrestrial mammal species present in New Zealand. Electrophoresis.

[6] Hrovatin K., Kunej T. (2018). Genetic sex determination assays in 53 mammalian species: Literature analysis and guidelines for reporting standardization. Ecol. Evol..

[7] Bilton T.P., Chappell A.J., Clarke S.M., Brauning R., Dodds K.G., McEwan J.C., Rowe S.J. (2019). Using genotyping-by-sequencing to predict gender in animals. Anim. Genet..

[8] Mori C., Matsumura S. (2021). Current issues for mammalian species identification in forensic science: A review. Int. J. Legal Med..

[9] Poetsch M., Seefeldt S., Maschke M., Lignitz E. (2001). Analysis of microsatellite polymorphism in red deer, roe deer, and fallow deer possible employment in forensic applications. Forensic Sci. Int..

[10] Eliason S. (2012). From the King’s deer to a capitalist commodity: A social historical analysis of the poaching law. Int. J. Comp. Appl..

[11] Szabolcsi Z., Egyed B., Zenke P., Padar Z., Borsy A., Steger V., Pasztor E., Csanyi S., Buzas Z., Orosz L. (2014). Constructing STR Multiplexes for Individual Identification of Hungarian Red Deer. J. Forensic Sci..

[12] Iyengar A. (2014). Forensic DNA analysis for animal protection and biodiversity conservation: A review. J. Nat. Conserv..

[13] Zenke P., Egyed B., Padar Z. (2017). Wildlife protection: Demonstrability of Wildlife crime with forensic DNA analysis. Casework applications. Hung. Vet. J..

[14] Morf N.V., Kopps A.M., Nater A., Lendvay B., Vasiljevic N., Webster L.M.I., Fautley R.G., Ogden R., Kratzer A. (2021). STRoe deer: A validated forensic STR profiling system for the European roe deer (*Capreolus capreolus*). Forensic Sci. Int..

[15] Hamlin B.C., Meredith E.P., Rodzen J., Strand J.M. (2021). OdoPlex: An STR multiplex panel optimized and validated for forensic identification and sex determination of North American mule deer (*Odocoileus hemionus*) and white-tailed deer (*Odocoileus virginianus*). Forensic Sci. Int..

[16] Bana N.Á., Nyiri A., Nagy J., Frank K., Nagy T., Stéger V., Schiller M., Lakatos P., Sugár L., Horn P. (2018). The red deer Cervus elaphus genome CerEla1.0: Sequencing, annotating, genes, and chromosomes. Mol. Genet. Genom..

[17] Sim Z., Monderman L., Hildebrand D., Packer T., Jobin R.M. (2021). Development and implementation of a STR based forensic typing system for moose (*Alces alces*). Forensic Sci. Int. Genet..

[18] Hungarian Ministry of Agriculture 79/2004. (V. 04.) FVM Order. https://njt.hu/jogszabaly/2004-79-20-82.

[19] Eurohunting Hunting Organization LTD. Hunting in Hungary. Hunting Seasons..

[20] Északerdő P.L.C. Hunting Tourism. http://www.eszakerdo.hu/angol/menu/vadinfo_eng.html/.

[21] Queirós J., Gortázar C., Alves P.C. (2020). Deciphering Anthropogenic Effects on the Genetic Background of the Red Deer in the Iberian Peninsula. Front. Ecol. Evol..

[22] Gouda S., Kerry R.G., Das A., Chauhan N.S. (2020). Wildlife forensics: A boon for species identification and conservation implications. Forensic Sci. Int..

[23] Linacre A. (2021). Animal Forensic Genetics. Genes.

[24] Smart U., Cihlar J.C., Budowle B. (2021). International Wildlife Trafficking: A perspective on the challenges and potential forensic genetics solutions. Forensic Sci. Int. Genet..

[25] Pfeiffer I., Brenig B. (2005). X- and Y-chromosome specific variants of the amelogenin gene allow sex determination in sheep (*Ovis aries*) and European red deer (*Cervus elaphus*). BMC Genet..

[26] Han S.H., Cho I.C., Lee S.S., Tandang L., Lee H., Oh H.S., Kim B.S., Oh M.Y. (2007). Identification of Species and Sex of Korean Roe Deer (*Capreolus pygargus* tianschanicus) Using SRY and CYTB Genes. Integr. Biosci..

[27] Qiao Y., Zou F., Wei K., Yue B. (2007). A Rapid Sex-Identification Test for the Forest Musk Deer (*Moschus berezovskii*) Based on the ZFX/ZFY Gene. Zool. Sci..

[28] Kim B.J., Lee Y.S., An J., Park H., Okumura H., Lee H., Min M. (2008). Species and sex identification of the Korean goral (*Nemorhaedus caudatus*) by molecular analysis of non-invasive samples. Mol. Cells.

[29] Barbosa A.M., Fernández-García J.L., Carranza J. (2009). A new marker for rapid Sex Identification of red deer (*Cervus Elaphus*). Hystrix It. J. Mamm..

[30] Gurgul A., Radko A., Słota E. (2010). Characteristics of X- and Y-chromosome specific regions of the amelogenin gene and a PCR-based method for sex identification in red deer (*Cervus elaphus*). Mol. Biol. Rep..

[31] Paul S., Ghosh T., Pandav B., Mohan D., Habib B., Nigam P., Mondol S. (2019). Rapid molecular assays for species and sex iden-tification of swamp deer and other coexisting cervids in human-dominated landscapes of the Terai region and upper Gangetic plains, northern India: Implications in understanding species distribution and population parameters. J. Genet..

[32] Linacre A., Gusmao L., Hecht W., Hellmann A.P., Mayr W.R., Parson W., Prinz M., Schneider P.M., Morling N. (2011). ISFG: Recommendations regarding the use of non-human (animal) DNA in forensic genetic investigations. Forensic Sci. Int. Genet..

[33] Amorim A. (2019). Nonhuman forensic genetics. Forensic Sci. Int. Genet..

[34] Wilson P.J., White B.N. (1998). Sex identification of elk (*Cervus elaphus* canadensis), moose (*Alces alces*), and white-tailed deer (*Odocoileus virginianus*) using the polymerase chain reaction. J. Forensic Sci..

[35] Cadamuro V.C., Bouakaze C., Croze M., Schiavinato S., Tonasso L., Ge’rard P., Fausser J.L., Gibert M., Dugoujon J.M., Braga J. (2015). Determined about sex: Sex-testing in 45 primate species using a 2Y/1X sex-typing assay. Forensic Sci. Int. Genet..

[36] Strah R., Kunej T. (2019). Molecular sexing assays in 114 mammalian species: In silico sequence reanalysis and a unified graphical visualization of diagnostic tests. Ecol. Evol..

[37] Johnson R.N., Wilson-Wilde L., Linacre A. (2014). Current and future directions of DNA in wildlife forensic science. Forensic Sci. Int. Genet..

[38] Meiklejohn K.A., Burnham-Curtis M.K., Straughan D.J., Giles J., Moore M.K. (2021). Current methods, future directions and considerations of DNA-based taxonomic identification in wildlife forensics. Forensic Sci. Int..

[39] Kanthaswamy S. (2015). Review: Domestic animal forensic genetics biological evidence, genetic markers, analytical approaches and challenges. Anim. Genet..

[40] Kurland J., Pires S.F., McFann S.C., Moreto W.D. (2017). Wildlife crime: A conceptual integration, literature review, and methodological critique. Crime Sci..

[41] Gudmannsson P., Berge J., Druid H., Ericsson G., Eriksson A. (2018). A Unique Fatal Moose Attack Mimicking Homicide. J. Forensic Sci..

[42] Shadrach B., Commane M., Hren C., Warshawsky I. (2004). A Rare Mutation in the Primer Binding Region of the Amelogenin Gene Can Interfere with Gender Identification. J. Mol. Diagn..

[43] Pajares G., Álvarez I., Fernández I., Pérez-pardal L., Goyache F., Royo L.J. (2007). A sexing protocol for wild ruminants based on PCR amplification of amelogenin genes AMELX and AMELY (short communication). Arch. Tierz. Dummerstorf.

[44] Frank K., Bana N.Á., Bleier N., Sugár L., Nagy J., Wilhelm J., Kálmán Z., Barta E., Orosz L., Horn P. (2020). Mining the red deer genome (CerEla1.0) to develop X-and Y-chromosome-linked STR markers. PLoS ONE.

[45] Tobe S.S., Linacre A. (2010). DNA typing in wildlife crime: Recent developments in species identification. Forensic Sci. Med. Pathol..

[46] Parkanyi V., Ondruska L., Vasicek D., Slamecka J. (2013). Multilevel D-loop PCR identification of hunting game. Appl. Transl. Genom..

[47] Druml B., Hochegger R., Cichna-Markl M. (2015). Duplex real-time PCR assay for the simultaneous determination of the roe deer (*Capreolus capreolus*) and deer (sum of fallow deer, red deer and sika deer) content in game meat products. Food Control.

[48] Ramón-Laca A., Gleeson D., Yockney I., Perry M., Nugent G., Forsyth D.M. (2014). Reliable discrimination of 10 ungulate species using high resolution melting analysis of faecal DNA. PLoS ONE.

[49] Mei M., Chen R., Gao X., Cao Y., Weng W., Duan Y., Tan X., Liu Z. (2020). Establishment and application of a 10-plex liquid bead array for the simultaneous rapid detection of animal species. J. Sci. Food Agric..

[50] Kaltenbrunner M., Hochegger R., Cichna-Markl M. (2018). Tetraplex real-time PCR assay for the simultaneous identification and quantification of roe deer, red deer, fallow deer and sika deer for deer meat authentication. Food Chem..

[51] The Scientific Working Group on DNA Analysis Methods (SWGDAM), SWGDAM Validation Guidelines for DNA Analysis Methods Approved 12/05/2016. https://www.swgdam.org/publications.

[52] Lucy M.I., SWFS Technical Working Group (2018). Standards and Guidelines for Wildlife Forensic Analysis.

[53] Marshall H.D., Johnstone K.A., Carr S.M. (2007). Species-specific oligonucleotides and multiplex PCR for forensic discrimination of two species of scallops, Placopecten magellanicus and Chlamys islandica. Forensic Sci. Int..

[54] Kim M.J., Lee Y.M., Suh S.M., Kim H.Y. (2020). Species Identification of Red Deer (*Cervus elaphus*), Roe Deer (*Capreolus capreolus*), and Water Deer (*Hydropotes inermis*) Using Capillary Electrophoresis-Based Multiplex PCR. Foods.

[55] Koehler A.V., Zhang Y., Wang T., Haydon S.R., Gasser R.B. (2020). Multiplex PCRs for the specific identification of marsupial and deer species from faecal samples as a basis for non-invasive epidemiological studies of parasites. Parasit. Vectors.

[56] Ogden R., Dawnay N., McEwing R. (2009). Wildlife DNA forensics—Bridging the gap between conservation genetics and law enforcement. Endanger. Species Res..

[57] Yang F., Ding F., Chen H., He M., Zhu S., Ma X., Jiang L., Li H. (2018). DNA Barcoding for the Identification and Authentication of Animal Species in Traditional Medicine. Evid. Based Complement Alternat. Med..

[58] Kaltenbrunner M., Hochegger R., Cichna-Markl M. (2022). Design of Mismatch Primers to Identify and Differentiate Closely Related (Sub)Species: Application to the Authentication of Meat Products. Methods Mol. Biol..

[59] Iacolina L., Corlatti L., Buzan E., Safner T., Šprem N. (2019). Hybridisation in European ungulates: An overview of the current status, causes, and consequences. Mam. Rev..

[60] Świsłocka M., Czajkowska M., Matosiuk M., Saveljev A.P., Ratkiewicz M., Borkowska A. (2019). No evidence for recent introgressive hybridization between the European and Siberian roe deer in Poland. Mamm. Biol..

[61] Šprem N., Stipoljev S., Ugarković D., Buzan E. (2021). First genetic analysis of introduced axis deer from Croatia. Mamm. Biol..

[62] Russell T., Cullingham C., Ball M., Pybus M., Coltman D. (2021). Extent and direction of introgressive hybridization of mule and white-tailed deer in western Canada. Evol. Appl..

[63] Štohlová Putnová L., Štohl R., Ernst M., Svobodová K. (2021). A Microsatellite Genotyping-Based Genetic Study of Interspecific Hybridization between the Red and Sika Deer in the Western Czech Republic. Animals.

[64] McDevitt A.D., Edwards C.J., O’Toole P., O’Sullivan P., O’Reilly C., Carden R.F. (2009). Genetic structure of, and hybridisation between, red (*Cervus elaphus*) and sika (*Cervus nippon*) deer in Ireland. Mamm. Biol..

[65] Smith S.L., Carden R.F., Coad B., Birkitt T., Pemberton J.M. (2014). A survey of the hybridisation status of Cervus deer species on the island of Ireland. Conserv. Genet..

[66] Smith S.L., Senn H.V., Pérez-Espona S., Wyman M.T., Heap E., Pemberton J. (2018). Introgression of exotic Cervus (nippon and canadensis) into red deer (*Cervus elaphus*) populations in Scotland and the English Lake District. Ecol. Evol..

[67] Fan H., Wang T., Li Y., Liu H., Dong Y., Zhang R., Wang H. (2021). Development and validation of a 1 K sika deer (*Cervus nippon*) SNP Chip. BMC Genom. Data.

[68] McFarlane S.E., Pemberton J.M. (2021). Admixture mapping reveals loci for carcass mass in red deer x sika hybrids in Kintyre, Scotland. G3 Bethesda.

[69] Amorim A., Pereira F., Alves C., Garcia O. (2020). Species assignment in forensics and the challenge of hybrids. Forensic Sci. Int. Genet..

[70] Lorenzini R., Fanelli R., Tancredi F., Siclari A., Garofalo L. (2020). Matching STR and SNP genotyping to discriminate between wild boar, domestic pigs and their recent hybrids for forensic purposes. Sci. Rep..

