# Supplementary material for: Molecular Sexing and Species Detection of Antlered European Hunting Game for Forensic Purposes
Source: Animals (Basel). 2022 Jan 20;12(3):246. doi: 10.3390/ani12030246 (PMC8833381; doi:10.3390/ani12030246)
Supplement: Supplementary file 1 [file animals-12-00246-s001.zip › animals-1511919-supplementary.pdf]

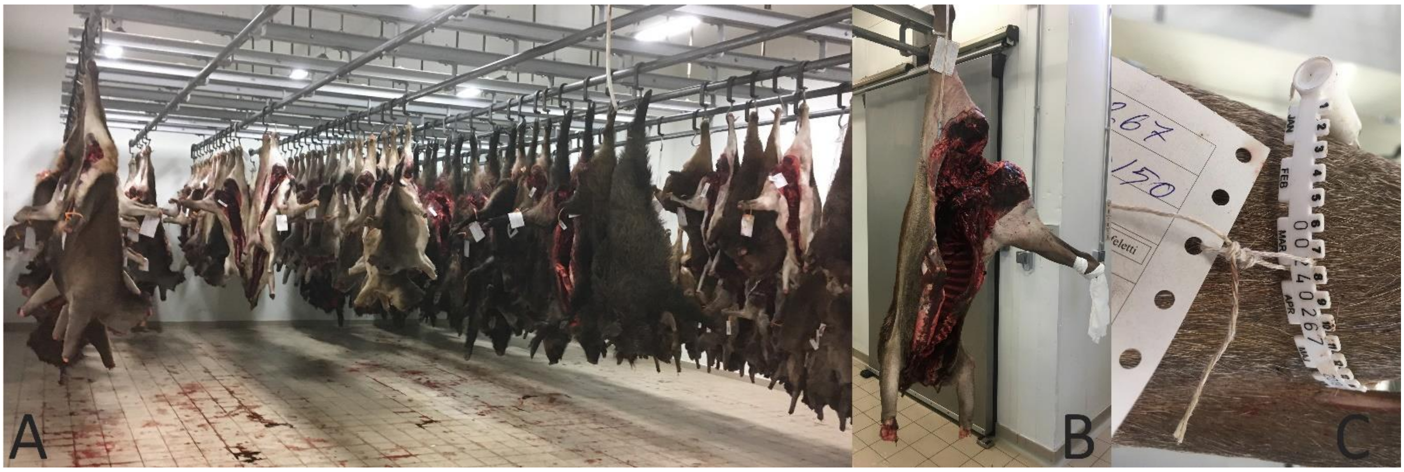

Figure S1. (A) Eviscerated and decapitated carcasses in processing house cold store. (B) Eviscerated and decapitated carcass of red deer, which was deliberately registered falsely as female. (C) Official tab of registered harvest

Table S1. Sample database from three antlered species (altogether n=115) used for validation studies, collected between 2019-2021 from different parts of Hungary [Pest County west (PesW); Pest County east (PesE); Békés County (Bek); Csongrád County (Cso), Hajdú-Bihar County (Haj), Tolna County (Tol), Fejér County (Fej), Veszprém County (Ves)]. Ce: red deer, Dd: fallow deer, Cc: roe deer

Sample code	Tissue type	Sex	Sample code	Tissue type	Sex	Sample code	Tissue type	Sex
Ce_Haj_1	hair	male	Dd_PesW_1	muscle	female	Cc_PesW_1	muscle	male
Ce_Ves_2	muscle	male	Dd_PesW_2	muscle	female	Cc_PesW_2	muscle	male
Ce_Ves_3	muscle	male	Dd_PesW_3	muscle	female	Cc_PesW_3	muscle	male
Ce_Ves_4	muscle	male	Dd_PesW_4	muscle	male	Cc_PesW_4	muscle	female
Ce_Ves_5	muscle	male	Dd_PesW_5	muscle	male	Cc_PesW_5	muscle	female
Ce_Ves_6	muscle	male	Dd_PesW_6	muscle	male	Cc_PesW_6	muscle	male
Ce_Ves_7	muscle	male	Dd_PesW_7	muscle	male	Cc_PesW_7	muscle	male
Ce_PesW_8	muscle	female	Dd_PesW_8	muscle	male	Cc_PesW_8	muscle	male
Ce_PesW_9	muscle	male	Dd_PesW_9	muscle	male	Cc_PesW_9	muscle	male
Ce_PesW_10	muscle	male	Dd_PesW_10	muscle	male	Cc_Cso_10	muscle	female
Ce_Zal_11	muscle	female	Dd_PesW_11	muscle	male	Cc_PesW_11	muscle	male
Ce_Zal_12	muscle	male	Dd_PesW_12	muscle	female	Cc_PesW_12	muscle	male
Ce_PesW_13	muscle	male	Dd_PesW_13	muscle	male	Cc_PesW_13	muscle	male
Ce_PesW_14	muscle	male	Dd_PesW_14	muscle	male	Cc_PesW_14	muscle	male
Ce_PesW_15	muscle	male	Dd_PesW_15	muscle	male	Cc_PesW_15	muscle	male
Ce_PesW_16	muscle	male	Dd_PesW_16	muscle	male	Cc_PesW_16	muscle	male
Ce_PesW_17	muscle	male	Dd_PesW_17	muscle	male	Cc_PesW_17	muscle	female
Ce_PesW_18	muscle	male	Dd_PesW_18	muscle	male	Cc_PesW_18	muscle	male
Ce_PesW_19	muscle	male	Dd_Fej_19	muscle	female	Cc_PesW_19	muscle	male
Ce_PesW_20	muscle	male	Dd_Fej_20	muscle	female	Cc_PesW_20	muscle	male
Ce_PesW_21	muscle	male	Dd_Fej_21	muscle	male	Cc_PesW_21	muscle	male
Ce_PesW_22	muscle	male	Dd_Fej_22	muscle	female	Cc_PesW_22	muscle	male
Ce_PesW_23	muscle	male	Dd_Fej_23	muscle	female	Cc_PesW_23	muscle	male
Ce_PesW_24	muscle	male	Dd_Fej_24	muscle	female	Cc_PesW_24	muscle	male
Ce_PesW_25	muscle	male	Dd_PesE_25	muscle	female	Cc_PesW_25	muscle	male
Ce_PesW_26	muscle	male	Dd_PesE_26	muscle	female	Cc_PesW_26	muscle	male
Ce_PesW_27	hair	male	Dd_PesE_27	muscle	male	Cc_PesW_27	muscle	male
Ce_Som_28	muscle	female	Dd_PesE_28	muscle	female	Cc_PesW_28	muscle	male
Ce_Bar_29	muscle	female	Dd_PesE_29	muscle	male	Cc_PesW_29	muscle	male
Ce_PesW_30	feces	male	Dd_PesE_30	muscle	female	Cc_PesW_30	muscle	male
			Dd_PesE_31	muscle	female	Cc_PesW_31	muscle	male
			Dd_PesE_32	muscle	female	Cc_PesW_32	muscle	male

Dd_PesE_33	muscle	female	Cc_PesW_33	muscle	male
Dd_PesE_34	muscle	female	Cc_PesW_34	feces	female
Dd_PesW_35	muscle	female	Cc_PesW_35	muscle	male
Dd_PesW_36	muscle	female	Cc_PesW_36	feces	male
Dd_PesW_37	muscle	female	Cc_Haj_37	muscle	female
Dd_PesW_38	muscle	male	Cc_PesW_38	feces	female
Dd_PesW_39	feces	male			
Dd_Tol_40	muscle	female			
Dd_PesE_41	muscle	male			
Dd_PesE_42	muscle	female			
Dd_PesE_43	muscle	female			
Dd_PesE_44	feces	male			
Dd_PesE_45	muscle	female			
Dd_PesE_46	muscle	female			
Dd_PesE_47	feces	female			

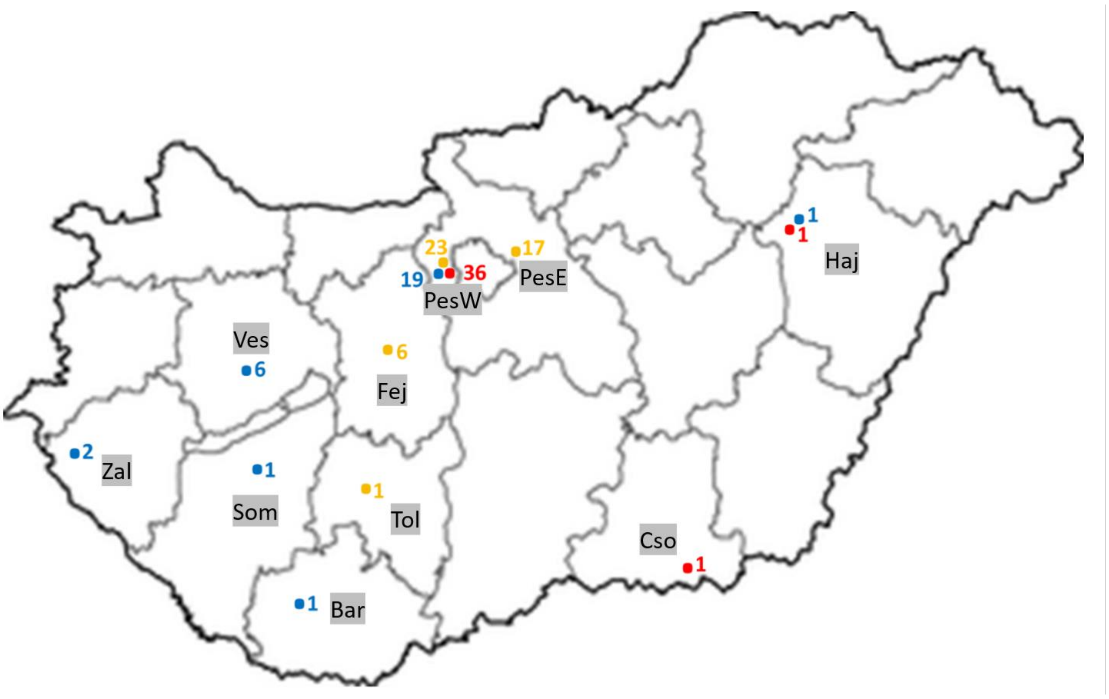

Figure S2. Distribution of collected samples within Hungary from three antlered species (altogether n=115) used for validation studies. Blue: red deer (n=30), yellow: fallow deer (n=47), red: roe deer (n=38)

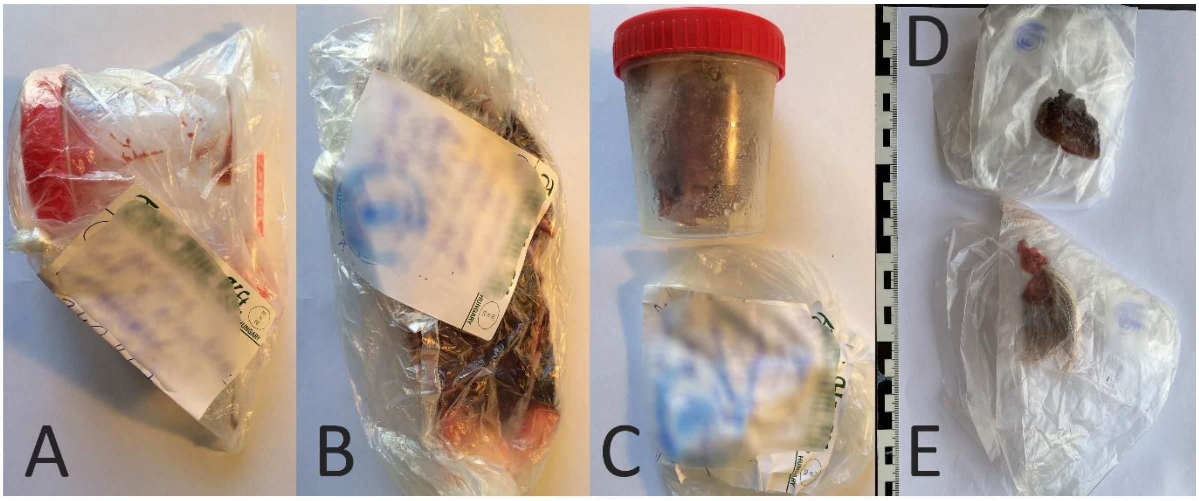

Figure S3. Field sample types. (A) blood and vessel, (B) bone / rib, (C) muscle, (D) muscle, (E) corium with hairs

Table S2. In silico intra-species variation study of developed primers, using different number of sequences downloaded from NCBI GenBank. Ce: red deer, Dd: fallow deer, Cc: roe deer. [a-m]: accession numbers and information about the downloaded sequences

Primer name (lenght of alignment in basepairs)	Number of sequences obtained from GenBank			Number of polymorphic sites			Max. number of polymorphic sites within a single sequence		
	Ce	Dd	Cc	Ce	Dd	Cc	Ce	Dd	Cc
AmelX/Y F (18)	3 [a]	2 [b]	2 [c]	0	0	0	0	0	0
AmelX/Y R (18)	6 [d]	2 [b]	2 [c]	0	0	1	0	0	1
SRY F (20)	22 [e]	1 [f]	5 [g]	0	0	0	0	0	0
SRY R (20)	22 [e]	1 [f]	5 [g]	0	0	0	0	0	0
Cytb Ce F (31)	435 [h]	-	-	9	-	-	4	-	-
Cytb Dd F (25)	-	4 [i]	-	-	0	-	-	0	-
Cytb Cc F (36)	-	-	135 [j]	-	-	3	-	-	3
Cytb R (26)	266 [k]	5 [l]	135 [j]	2	1	2	2	1	2

[a]: FJ946989, KJ542362, KJ542367; [b]: KJ542361, KJ542366; [c]: KJ542360, KJ542365; [d]: AY453391, FJ946989, FJ946990, KJ542362, KJ542367, XM043895580; [e]: DQ888682-96, EF062527-28, EF100133-35, EF693906-07; [f]: DQ888701; [g]: DQ888700, HG326979, KJ558344-46; [h]: all available cytb sequences from Cervus elaphus (taxid:9860) accessed on: 20.12.2021. containing Cytb Ce F primer binding region; [i]: AJ000022, JN632629, NC020700, MN746794; [j]:, KJ558288-333, KJ681491, KT964391, KT964393-94, KT964396-402, KT964404-09, KT964411-12, KT964414, KT964419-27, KT964431-42, KU845311, MT297579-624, Y14951; [k]: all available cytb sequences from Cervus elaphus (taxid:9860) accessed on: 20.12.2021. containing Cytb Ce R primer binding region; [l]: AJ000022, JN632629, KM224369, NC020700, MN746794;

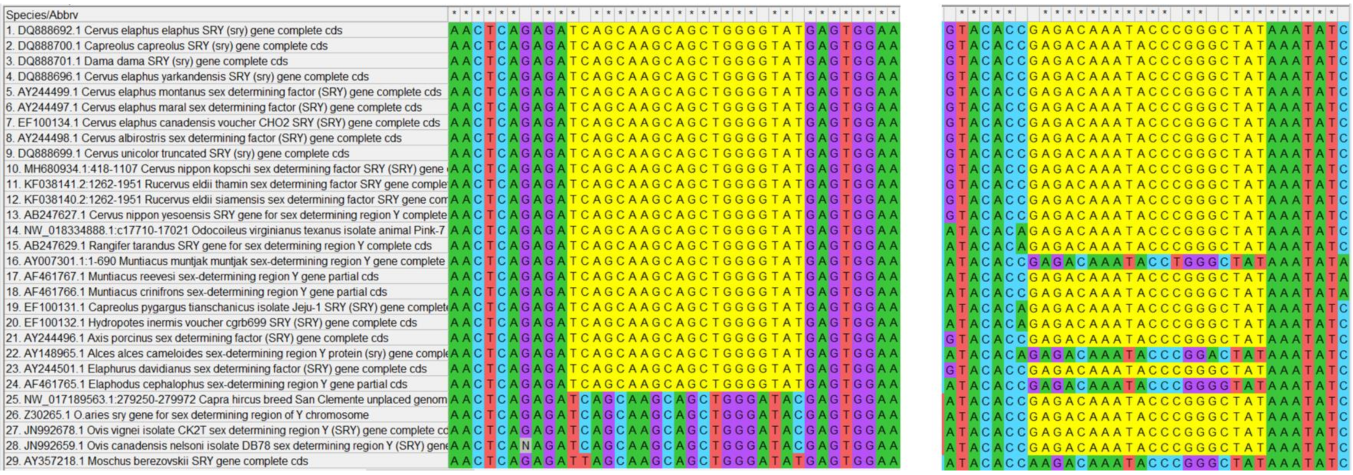

Figure S4. The forward (left side) and reverse (right side) primer binding regions marked out with yellow color within the highly conservative regions of the SRY gene identified among species by MEGA.

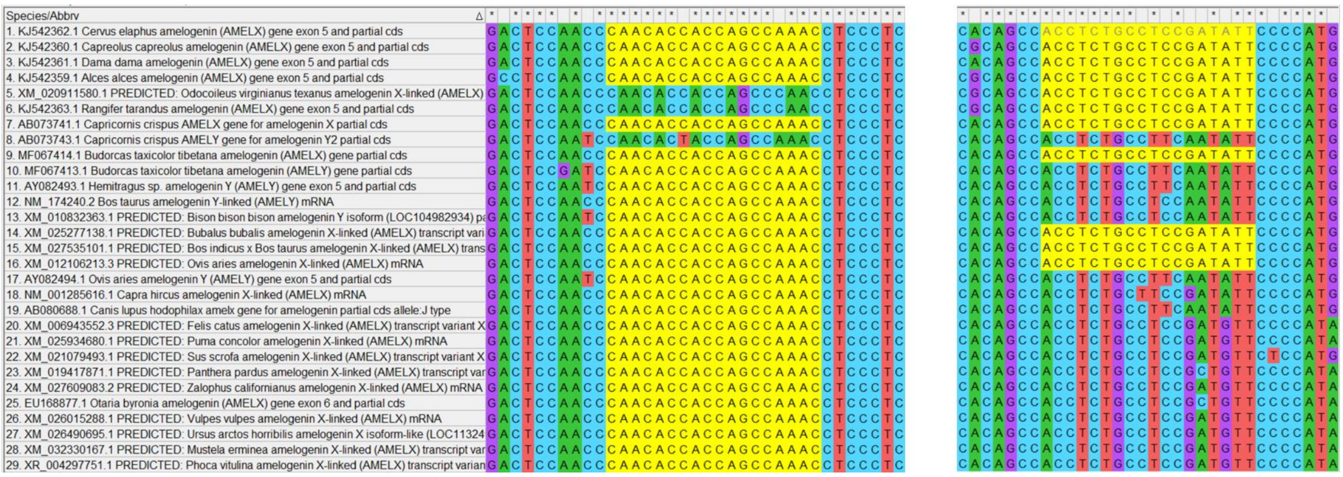

Figure S5. The forward (left side) and reverse (right side) primer binding regions marked out with yellow color within the highly conservative regions of the AmelogeninX/Y gene among species identified by MEGA.

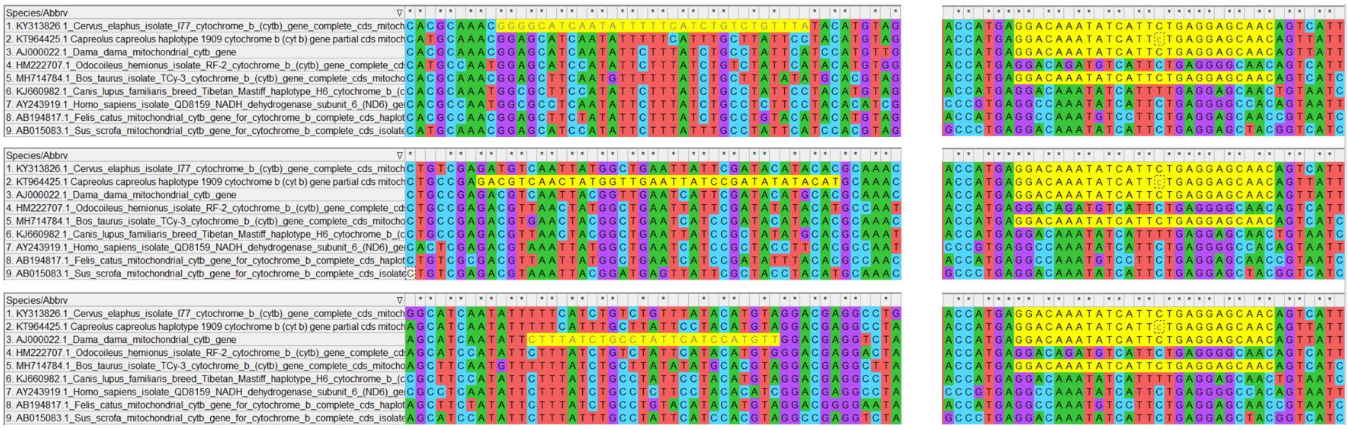

Figure S6. The species-specific forward (left side) and the universal reverse (right side) primer binding regions marked out with yellow color within the cytochrome b gene among species identified by MEGA.

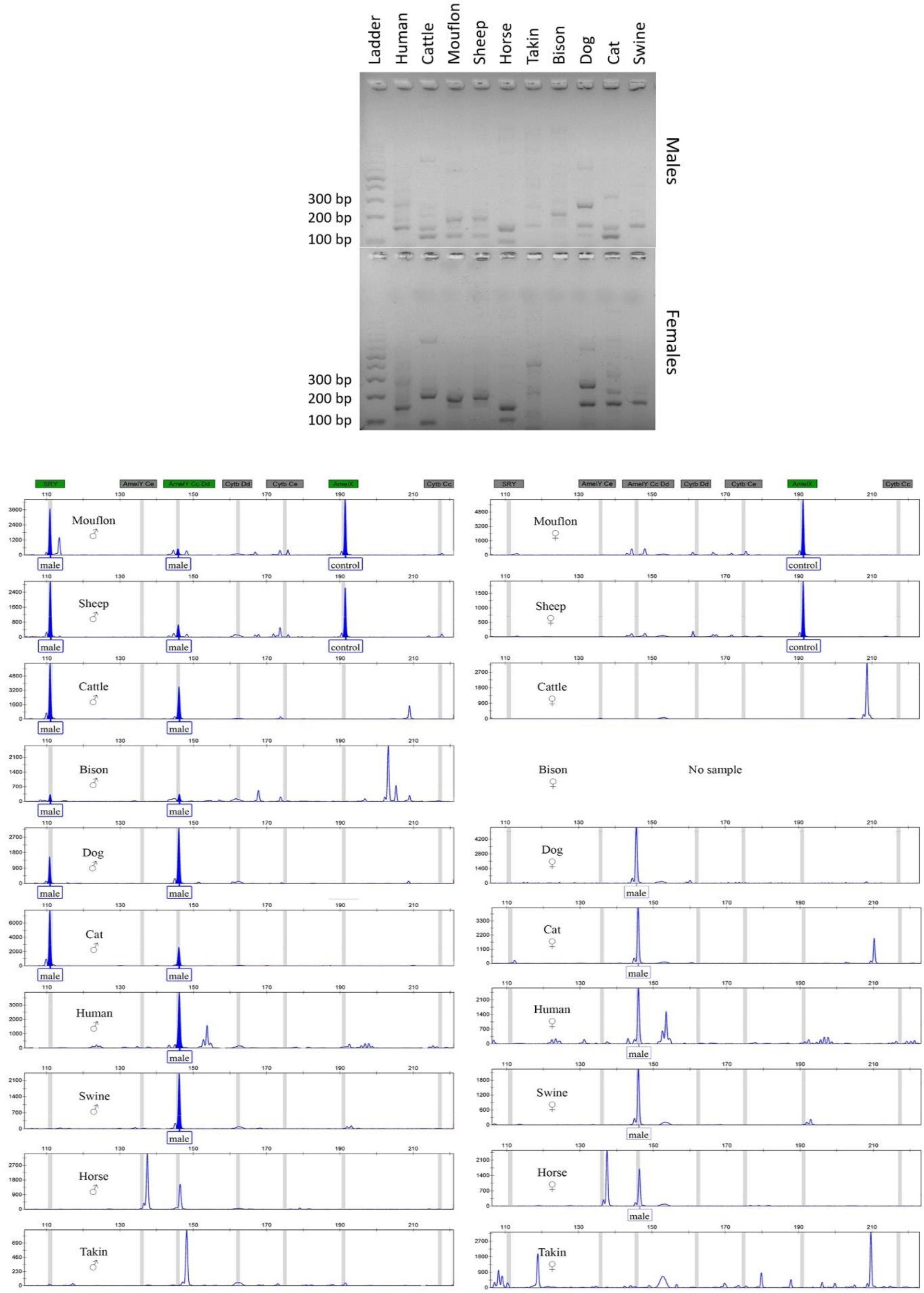

Figure S7. Species specificity tested on male and female samples using the *DeerSex-plex* assay, detected on agarose gel (at the top) and by capillary electrophoresis (below).

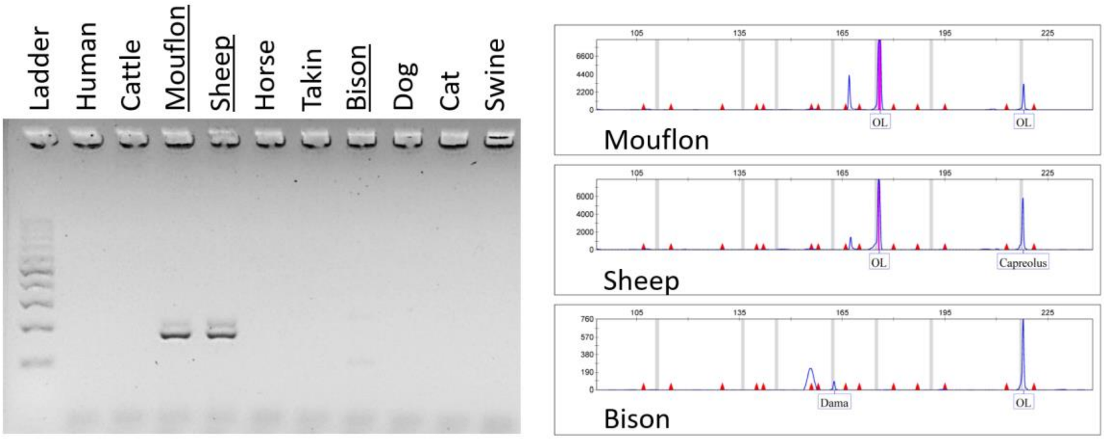

Figure S8. Species specificity tested on female samples using the *DeerSpec-plex* assay, detected by agarose gel and capillary electrophoresis.

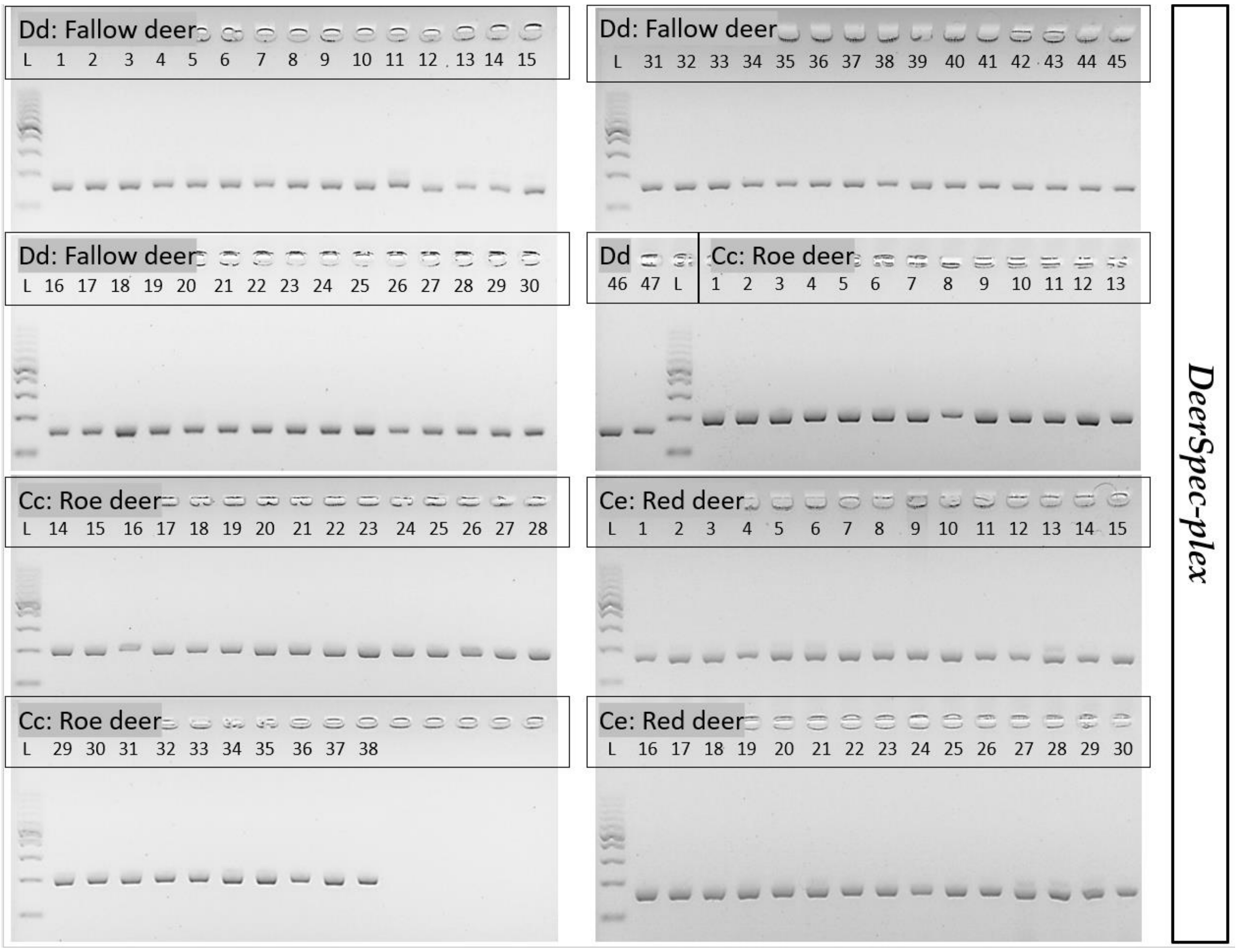

Figure S9. Intra-species specificity study: population samples from three species amplified with *DeerSpec-plex* and detected on agarose gel

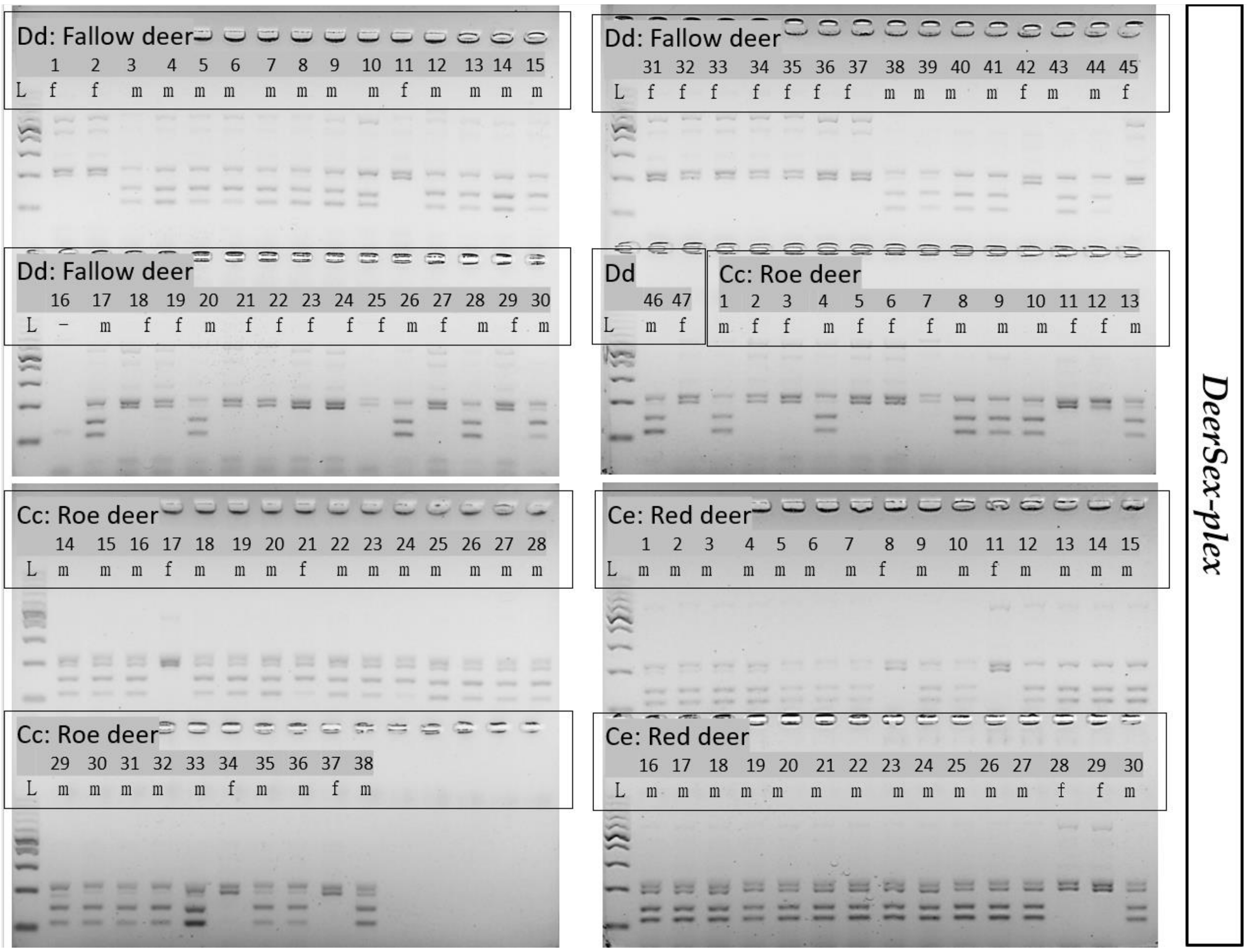

Figure S10. Intra-species specificity study: population samples (n=115) from three species amplified with *DeerSex-plex* and detected on agarose gel. m: male, f: female

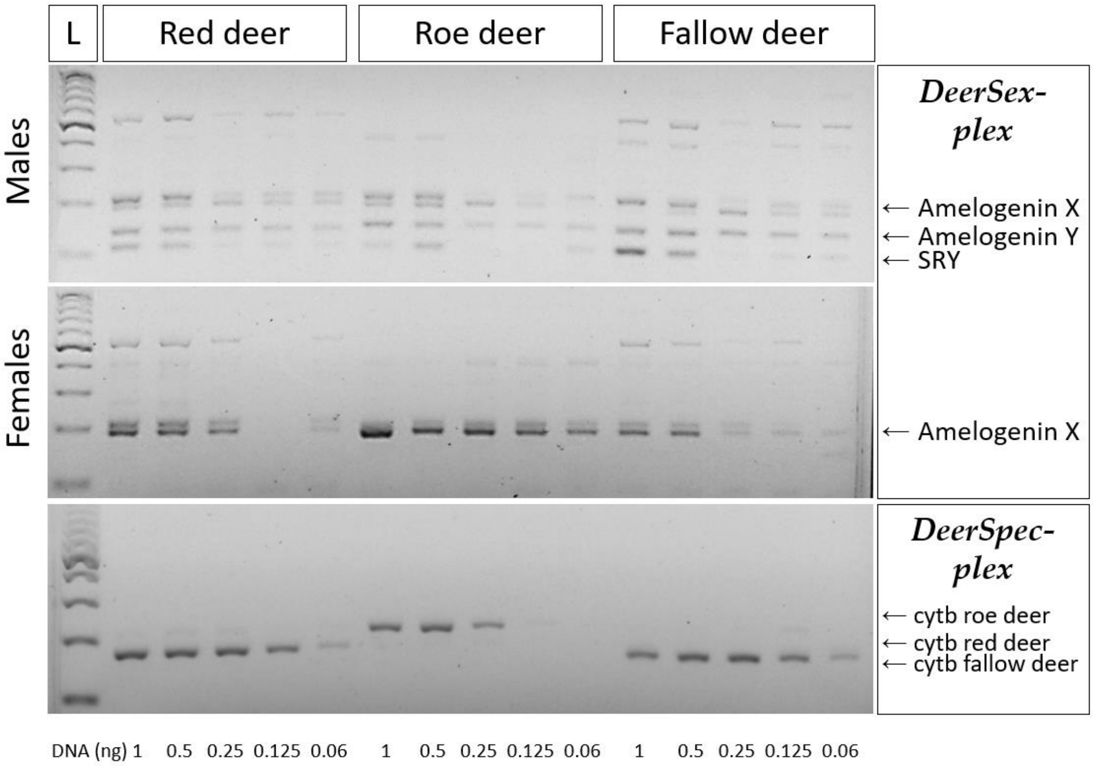

Figure S11. Sensitivity tested on male and female samples using the *DeerSex-plex* and *DeerSpec-plex* assays on agarose gel.

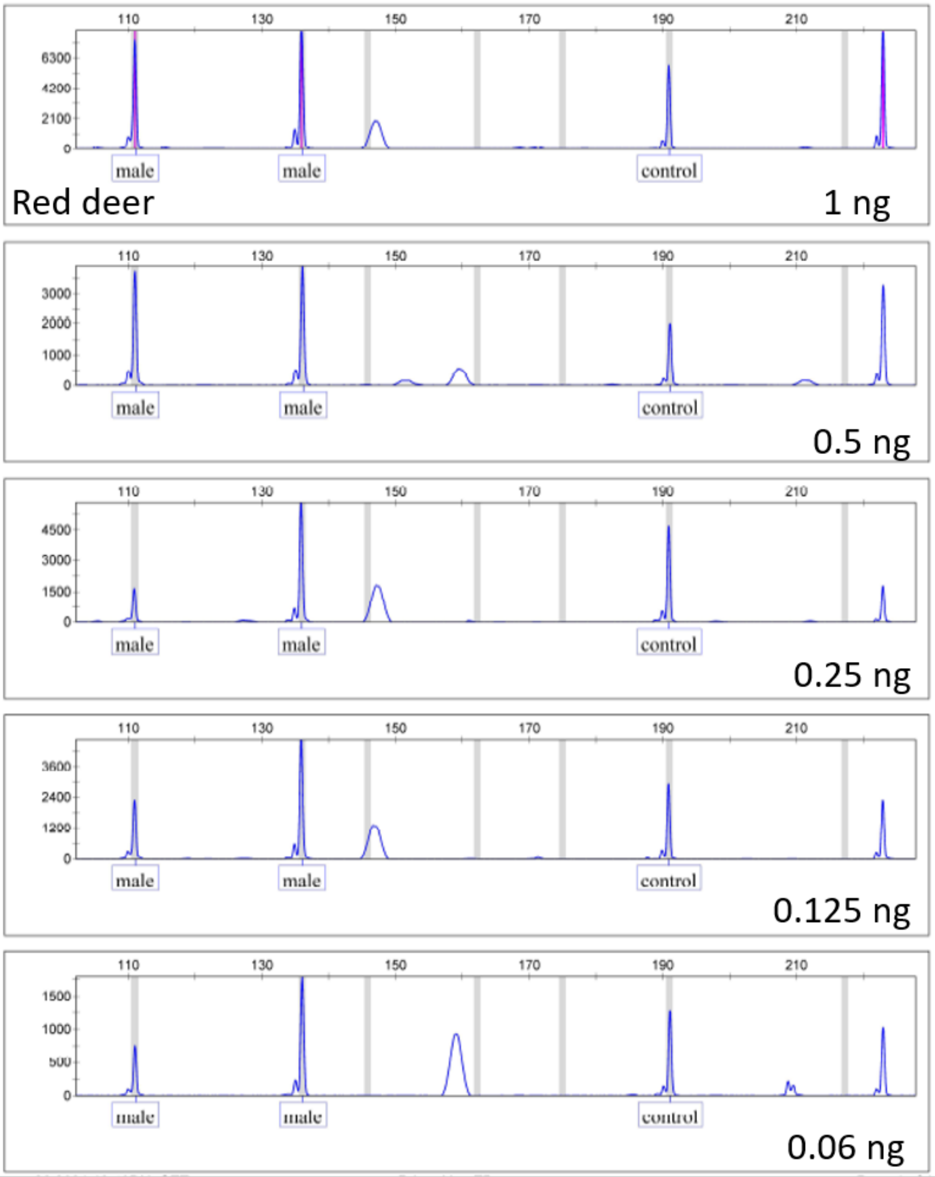

Figure S12a. The SRY and AMELX/Y loci in red deer samples amplified with varying amounts of input DNA with the DeerSex-plex assay. A dilution of the red deer DNA was performed, and 1.0, 0.5, 0.25, 0.125 or 0.06 ng of DNA (from the top down, respectively) were used in the amplification reaction.

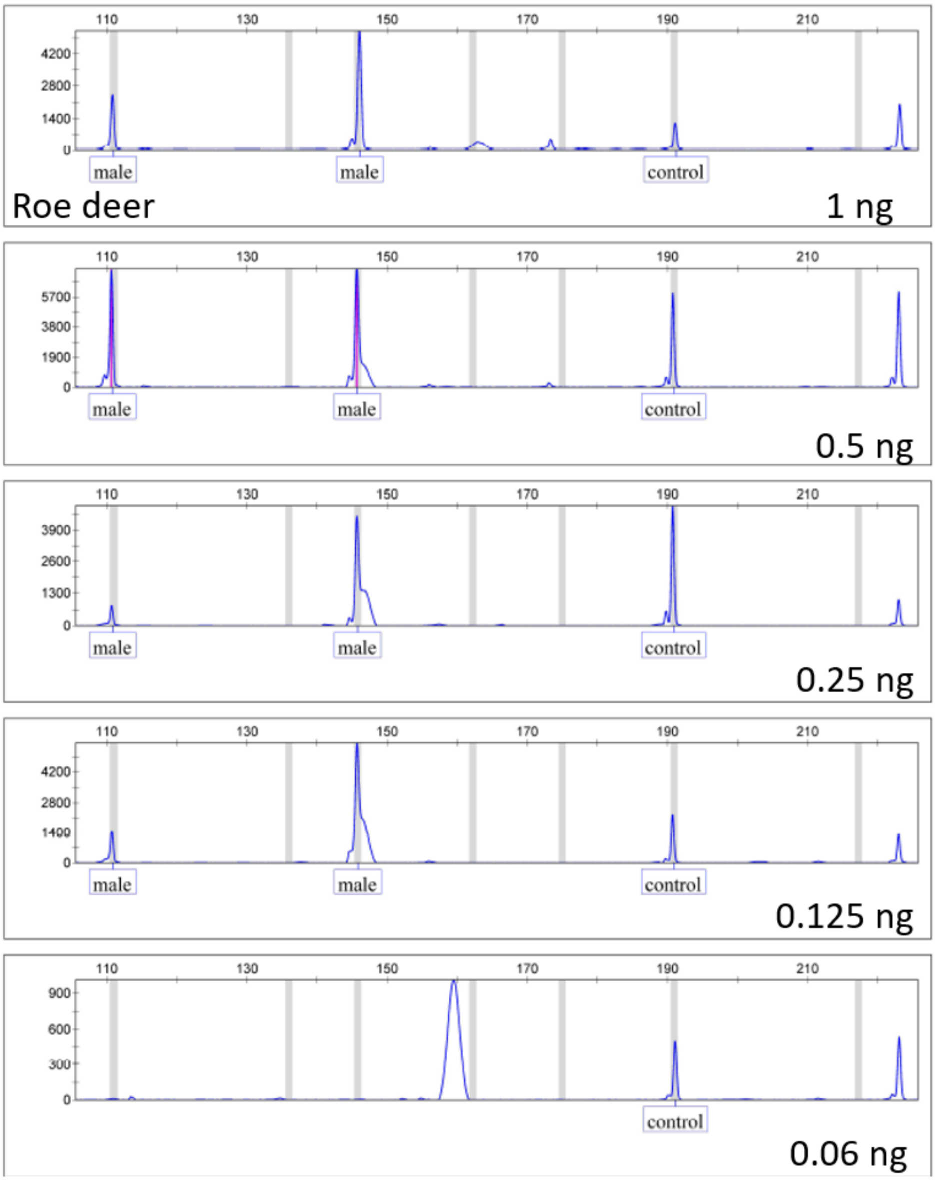

Figure S12b. The SRY and AMELX/Y loci in roe deer samples amplified with varying amounts of input DNA with the *DeerSex-plex* assay. A dilution of the roe deer DNA was performed, and 1.0, 0.5, 0.25, 0.125 or 0.06 ng of DNA (from the top down, respectively)were used in the amplification reaction. Allele drop-outs can be observed under 0.5 ng input DNA.

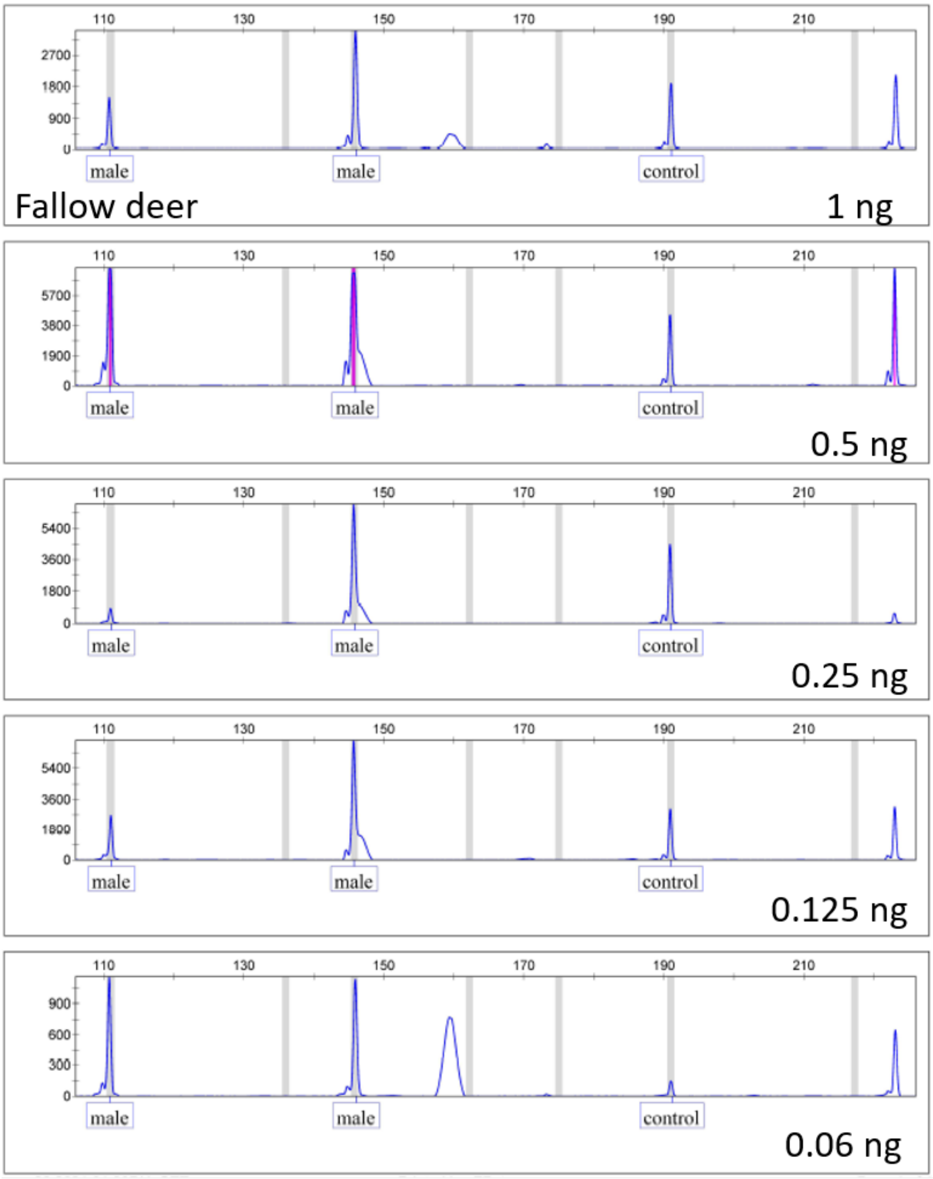

Figure S12c. The SRY and AMELX/Y loci in fallow deer samples amplified with varying amounts of input DNA with the *DeerSex-plex* assay. A dilution of the fallow deer DNA was performed, and 1.0, 0.5, 0.25, 0.125 or 0.06 ng of DNA (from the top down, respectively) were used in the amplification reaction.

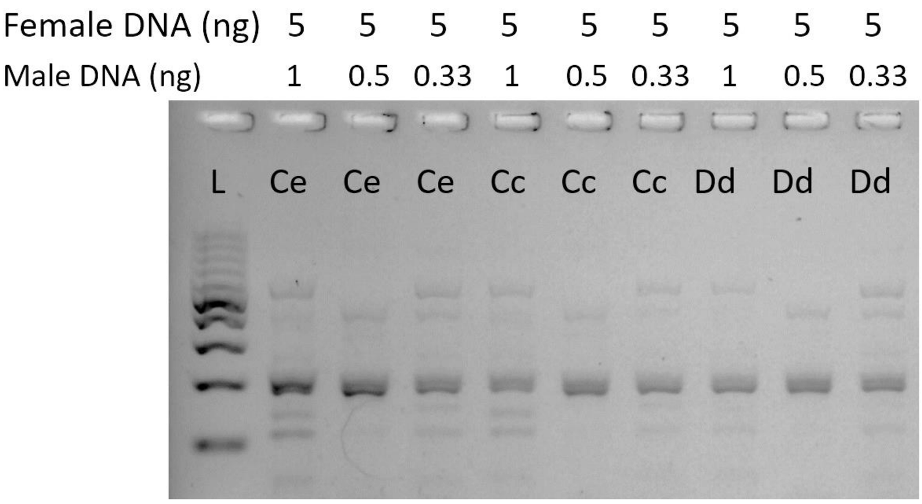

Figure S13. Mixture study: red deer (Ce), roe deer (Cc) and fallow deer (Dd) samples amplified with varying amounts of input male DNA with the *DeerSex-plex* assay.

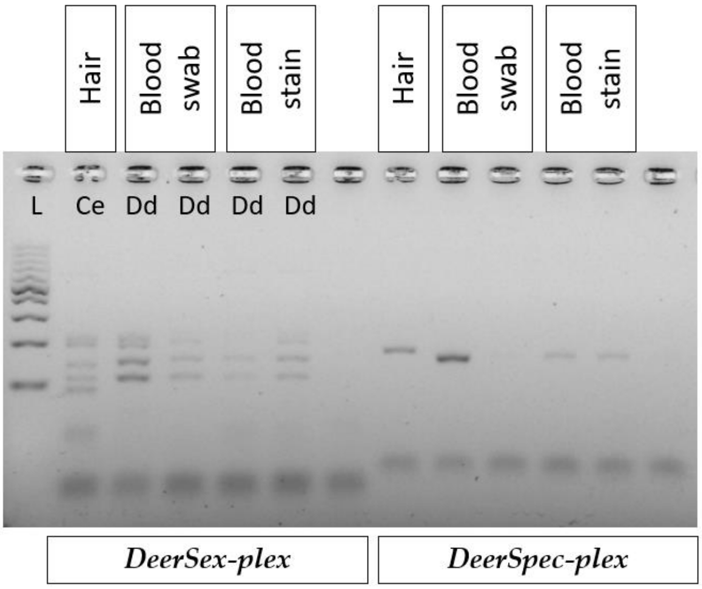

Figure S14. Case type samples amplified with both assays. Ce: red deer, Dd: fallow deer

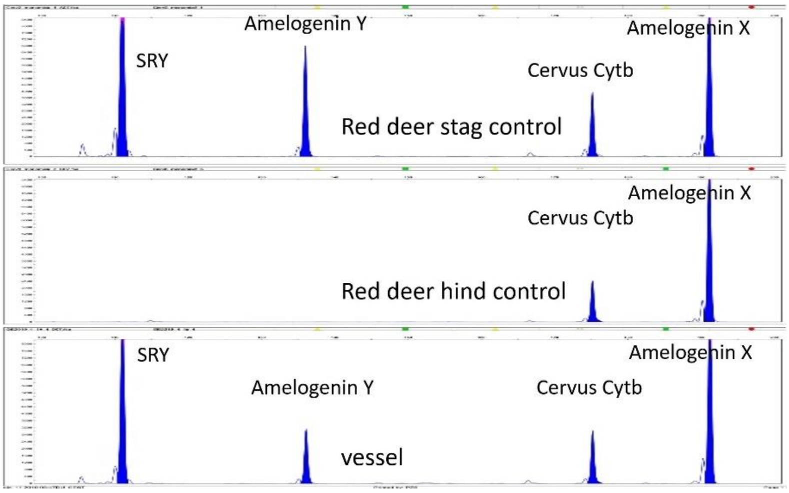

Figure S15. CE result of field sample (vessel) in mock case_1.

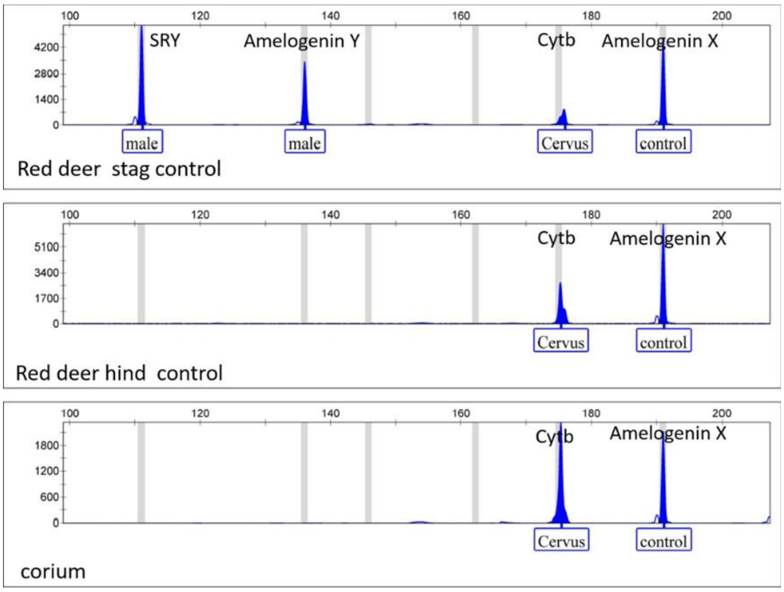

Figure S16. CE result of field sample (corium) in mock case_2.
